# From nappe stacking to exhumation: Cretaceous tectonics in the Apuseni Mountains (Romania)

**DOI:** 10.1007/s00531-016-1335-y

**Published:** 2016-05-11

**Authors:** Martin Kaspar Reiser, Ralf Schuster, Richard Spikings, Peter Tropper, Bernhard Fügenschuh

**Affiliations:** 1 Institut für Geologie, Universität Innsbruck, Innrain 52, Bruno Sander Haus, 6020 Innsbruck, Austria; 2 Geologische Bundesanstalt, Neulinggasse 38, 1030 Vienna, Austria; 3 Department of Mineralogy, University of Geneva, Rue des Maraîchers 13, office 42 (building B), 1211 Geneva 4, Switzerland; 4 Institut für Mineralogie und Petrologie, Universität Innsbruck, Innrain 52, Bruno Sander Haus, 6020 Innsbruck, Austria

**Keywords:** Geochronology, Cretaceous, Tectonics, Exhumation, Tisza, Dacia, Apuseni Mountains, Ar–Ar, Rb–Sr

## Abstract

New Ar–Ar muscovite and Rb–Sr biotite age data in combination with structural analyses from the Apuseni Mountains provide new constraints on the timing and kinematics of deformation during the Cretaceous. Time–temperature paths from the structurally highest basement nappe of the Apuseni Mountains in combination with sedimentary data indicate exhumation and a position close to the surface after the Late Jurassic emplacement of the South Apuseni Ophiolites. Early Cretaceous Ar–Ar muscovite ages from structurally lower parts in the Biharia Nappe System (Dacia Mega-Unit) show cooling from medium-grade conditions. NE–SW-trending stretching lineation and associated kinematic indicators of this deformation phase (D1) are overprinted by top-NW-directed thrusting during D2. An Albian to Turonian age (110–90 Ma) is proposed for the main deformation (D2) that formed the present-day geometry of the nappe stack and led to a pervasive retrograde greenschist-facies overprint. Thermochronological and structural data from the Bihor Unit (Tisza Mega-Unit) allowed to establish E-directed differential exhumation during Early–Late Cretaceous times (D3.1). Brittle detachment faulting (D3.2) and the deposition of syn-extensional sediments indicate general uplift and partial surface exposure during the Late Cretaceous. Brittle conditions persist during the latest Cretaceous compressional overprint (D4).

**Electronic supplementary material:**

The online version of this article (doi:10.1007/s00531-016-1335-y) contains supplementary material, which is available to authorized users.

## Introduction

**Fig. 1:**
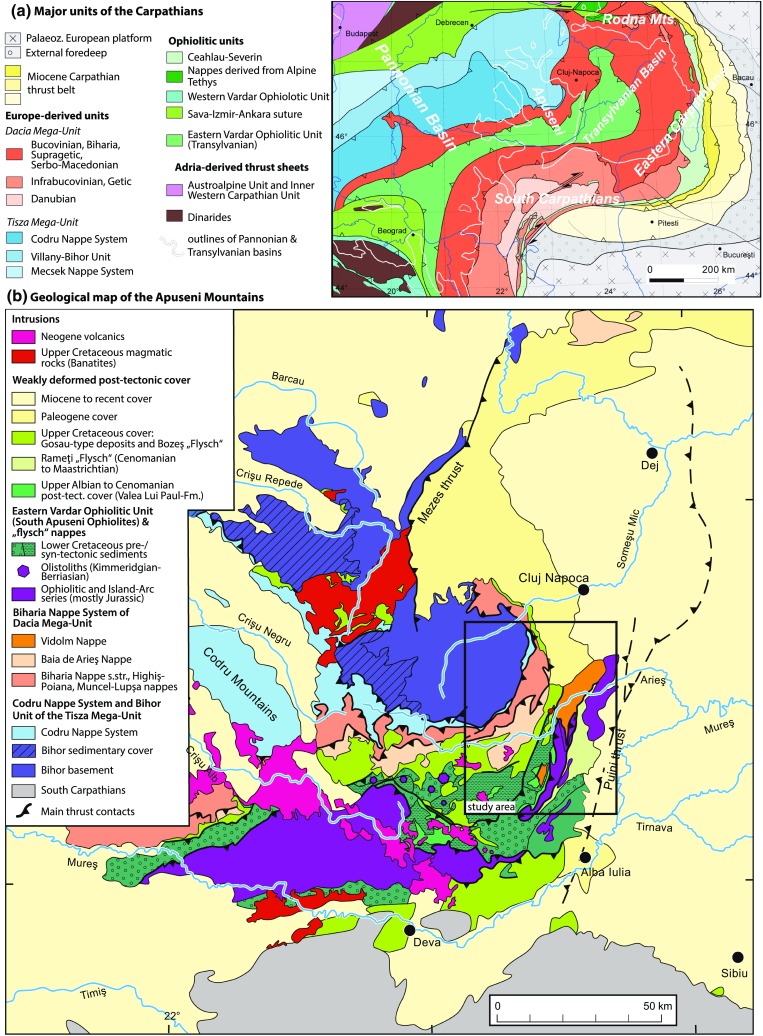
**a** Major tectonic units of the Carpathians according to correlation from Schmid et al. (2008). The Cenozoic cover sediments of the Pannonian and Transylvanian basins are not shown. **b** Geological map of the Apuseni Mountains. The study area is marked by a *black frame*. Modified from Kounov and Schmid (2013)

The Alpine–Carpathian–Dinaride orogenic system was the focus of several recent studies, which resulted in new concepts for the Alpine evolution of the region (e.g. Schmid et al. 2008; Ustaszewski et al. 2009; Dombrádi et al. 2010; Faccenna and Becker 2010; Handy et al. 2010; Merten 2011; Schefer 2012). The Apuseni Mountains in Romania take a central position within this system and expose the contact between the Tisza and Dacia Mega-Units (together with obducted ophiolites derived from the Neotethys ocean), which is otherwise largely covered by Cenozoic sediments of the Pannonian and Transylvanian basins (Fig. 1; Csontos and Vörös 2004; Schmid et al. 2008). This makes the Apuseni Mountains the ideal study area for the tectonometamorphic evolution of the Tisza and Dacia Mega-Units and allows for further constraints on the Alpine–Carpathian–Dinaride system (Csontos and Vörös 2004; Krézsek and Bally 2006; Schmid et al. 2008; Ustaszewski et al. 2009; Merten 2011). An ongoing controversy about the early Alpine tectonic evolution, the palaeogeographic situation, and tectonic position of the South Apuseni Ophiolites and flysch nappes (e.g. Ionescu et al. 2009; Hoeck et al. 2009; Pană 2010, and references therein) indicates that the evolution of the Alpine–Carpathian–Dinaride system of orogens is still not fully understood. One reason for the divergences in the geodynamic models is the fact that the early Alpine deformation events which shaped the Apuseni Mountains are still poorly studied in terms of their timing and kinematics of deformation (Kounov and Schmid 2013, p. 3). Recent studies focused on very low- and low-temperature thermochronology and allowed generating a detailed picture of the Cretaceous and Cenozoic thermotectonic evolution of the major tectonic units in the Apuseni Mountains (Merten et al. 2011; Kounov and Schmid 2013). However, with the exception of previously published $^{40}\mathrm{Ar}/^{39}\mathrm{Ar}$ data by Dallmeyer et al. (1999), other mid- to high-temperature thermochronological data are still missing. The large number of models which were published for the Mesozoic evolution of the Apuseni Mountains (e.g. Sǎndulescu 1984; Lupu et al. 1993; Csontos and Vörös 2004; Schuller 2004; Schmid et al. 2008; Hoeck et al. 2009; Ionescu et al. 2009; Kounov and Schmid 2013) testifies for the complexity of the study area. Limiting factors for all tectonic models are the relative scarcity of relevant exposures, the lack of stratigraphic control, and the polyphase metamorphic overprint during the Variscan and Alpine evolution. Through the integration of structural and thermochronological data, new and published data sets, and local and regional constraints, our study aims to provide a new model for the tectonic evolution of the Apuseni Mountains.

## Geological background

**Fig. 2:**
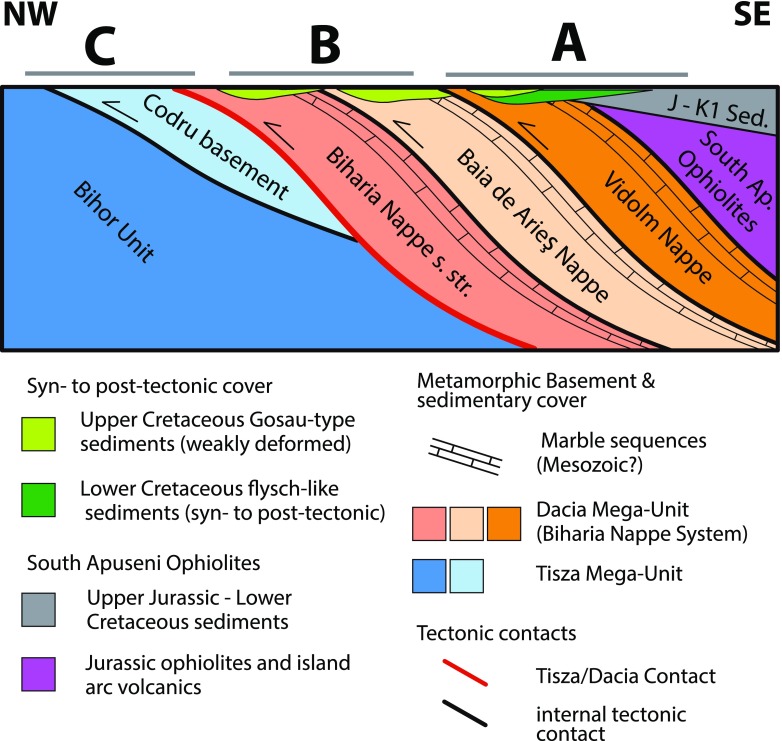
Schematic NW–SE-oriented cross section through the present-day nappe stack of the study area to illustrate the structural positions of the nappes with respect to each other. *Capital letters* (A, B, C) illustrate the relative structural position of the sectors used in Figs. 3, 4, and 11. Modified from Kounov and Schmid (2013)

The Apuseni Mountains mainly comprise pre-Variscan, polyphase metamorphic crystalline basement, Palaeozoic granitoid intrusions, Upper Palaeozoic cover, Jurassic ophiolites, and Mesozoic sequences of variable thickness (e.g. Ianovici et al. 1976; Kräutner 1980; Bleahu et al. 1981; Sǎndulescu 1984; Pană 1998; Dallmeyer et al. 1999; Balintoni et al. 2009, 2010). Following the pre-Mesozoic tectonics, the units experienced polyphase nappe stacking, metamorphic overprinting, and deformation during Alpine orogeny (Balintoni 1994; Dallmeyer et al. 1999). Cretaceous syn- to post-tectonic sedimentary sequences cover the previously stacked nappes (Bleahu et al. 1981; Ellero et al. 2002; Schuller 2004; Kounov and Schmid 2013). An important aspect of the Apuseni Mountains is the exposed contact between the Tisza and Dacia Mega-Units (see Fig. 1; Sǎndulescu 1994; Haas and Péró 2004; Csontos and Vörös 2004; Schmid et al. 2008; Kounov and Schmid 2013). Biogeographic data (e.g. Vörös 1977, 1993) indicate a neighbouring position of the Tisza and Dacia Mega-Units along the European continental margin during the Triassic and Early Jurassic. From the Middle Jurassic onwards, both units were separated from Europe due to the westward propagating opening of the Piemont-Ligurian ocean. Both mega-units experienced a significant change in fossil assemblage during the Bathonian, which indicates that they had come under the influence of the Adriatic palaeobiogeographic realm (e.g. Vörös 1977, 1993; Lupu 1984; Haas and Péró 2004). Palaeomagnetic data show a common apparent polar wander (APW) path for the European plate and the Tisza Mega-Unit up into the Cretaceous, 130 Ma ago (Hauterivian/Barremian; Martón 2000). Between Campanian and mid-Miocene times, northward displacement and clockwise rotation by some 80°–90° affected both Tisza and Dacia Mega-Units (e.g. Pătrascu et al. 1990, 1994; Martón et al. 2007; Panaiotu and Panaiotu 2010). The Tisza Mega-Unit is a large composite structural unit comprising three main nappe systems of Alpine origin: Mecsek (including the Szolnok Unit), Villány-Bihor (Bihor), and Békés-Codru, referred to as Codru (Fülöp 1994; Lelkes-Felvári et al. 2003; Haas and Péró 2004). In the Apuseni Mountains, the Tisza Mega-Unit only comprises the Bihor Unit and the Codru Nappe System according to Schmid et al. (2008) (Figs. 1, 2). Basement outcrops of the Tisza Mega-Unit are located in the Slavonian Mountains (Croatia), in the South Transdanubian ranges (Mecsek and Villány Hills; Hungary) and in the Apuseni Mountains (Romania). It was assumed that Alpine metamorphism of the Tisza Mega-Unit was only of retrogressive nature. However, modelling of monazite ages and petrographical relationships from the Slavonian Mountains (Balen et al. 2013; Balen 2014) show Early Cretaceous prograde Alpine metamorphism (113 ± 20 Ma) followed by a Late Cretaceous (82 ± 23 Ma) low-grade low-pressure metamorphism. P–T studies and geochronological data from boreholes in SE Hungary (contact area between the Tisza and Dacia Mega-Units) provide evidence for Alpine prograde amphibolite-facies metamorphism (650–680 $^{\circ }\mathrm {C}$ and 5–6 GPa, reaching up to 9 GPa in some samples) accompanied by penetrative mylonitization and followed by a continuous decrease in temperature and pressure conditions (Árkai et al. 2000; Árkai 2001; Horváth and Árkai 2002; Lelkes-Felvári et al. 2003, 2005). The Dacia Mega-Unit, also known as Internal Dacides (Sǎndulescu 1984, 1994), represents the most internal tectonic unit of the East and South Carpathians. According to the correlation by Schmid et al. (2008), the “Europe-derived units” of the Dacia Mega-Unit include the Biharia, Getic, Danubian, and Bucovinian Nappe Systems, which can be found in the Rhodopes, Dinarides, South Carpathians, Central East Carpathians, and the Apuseni Mountains. The Dacia Mega-Unit comprises pre-Variscan metasedimentary and metaigneous rocks (Balintoni et al. 2010) and isolated, incomplete Triassic to Jurassic sedimentary sequences covered by post-tectonic late Albian and/or younger deposits (e.g. Bleahu et al. 1981; Csontos and Vörös 2004; Schuller et al. 2009; Kounov and Schmid 2013). Based on the fact that the Transylvanian Ophiolitic unit (which includes the South Apuseni Ophiolites; Hoeck et al. 2009; Ionescu et al. 2009, 2010) overlies the Bucovinian Nappe System and the Biharia Nappe System (cf. Sǎndulescu 1984; Krézsek and Bally 2006), Schmid et al. (2008) attributed the Biharia Nappe System to the Dacia Mega-Unit, while other authors (e.g. Pană 1998; Csontos and Vörös 2004; Haas and Péró 2004) previously considered it to be an integral part of Tisza.

### Tectonic evolution of the Apuseni Mountains

During the Alpine orogeny, five tectonic events were inferred in the Apuseni Mountains (e.g. Ianovici et al. 1976; Bleahu et al. 1981; Sǎndulescu 1984; Balintoni and Iancu 1986; Balintoni 1994; Balintoni and Puşte 2002; Schuller 2004; Merten et al. 2011; Kounov and Schmid 2013): Late Jurassic emplacement of the South Apuseni Ophiolites, Early Cretaceous nappe stacking (125–100 Ma; “Austrian Phase”), Early–Late Cretaceous nappe stacking (93–89 Ma; “Turonian Phase”), Late Cretaceous extension related to deposition of syn- to post-tectonic sediments (Lupu and Lupu 1962; Schuller 2004), and latest Late Cretaceous–Early Palaeogene compression (70–55 Ma; “Laramian Phase”). The present-day nappe stack with the Bihor Unit as the lowest unit, overlain by the Codru Nappe System and the Biharia Nappe System, was completed by the Turonian phase (Fig. 2; Kounov and Schmid 2013). The highest nappe of the Biharia Nappe System (Vidolm Nappe) is overlain by the South Apuseni Ophiolites and their Jurassic–Early Cretaceous cover. The nappe stack displays a general dip towards the SE (Fig. 2). Note that all directions will be given in present-day coordinates in spite of the Palaeogene–Neogene clockwise rotation of the Tisza and Dacia Mega-Units into the Carpathian embayment (approximately 90$^{\circ }$; Martón et al. 2007).

#### Late Jurassic obduction

During the Late Jurassic, in the course of the subducting Neotethys Ocean, the South Apuseni Ophiolites were thrust/obducted on top of the Biharia Nappe System (Burchfiel 1980; Schmid et al. 2008; Kounov and Schmid 2013). Following the correlation of Schmid et al. (2008) and according to geochemical data of Bortolotti et al. (2002; 2004), the South Apuseni Ophiolites belong to the East Vardar Ophiolitic Unit. The direction of obduction, the palaeogeographic position of the Dacia Mega-Unit, and more specifically the attribution of the ophiolites to a northern (Piemont-Liguria) or southern (Neotethys) oceanic realm are still a matter of debate (cf. Sǎndulescu 1984; Haas and Péró 2004; Csontos and Vörös 2004; Schmid et al. 2008; Ionescu et al. 2009; Hoeck et al. 2009; Schuller et al. 2009; Kounov and Schmid 2013). No metamorphic sole, nor associated Jurassic ophiolitic mélanges have been reported so far from this group of ophiolite-bearing nappes, but Savu (2007, and references therein) reported burial metamorphism at temperatures from 200–400 $^{\circ }{\mathrm{C}}$ in the presumably Mesozoic sedimentary succession on top of the Vidolm Nappe. Limestones in the hanging wall are recrystallized, and psephites are mapped at the thrust contact (Balintoni et  al. 1987). An age of 168 ± 5 Ma (K/Ar whole-rock radiometric data; Nicolae et al. 1992), interpreted as formation age, was published for these MORB-type ophiolites, and two gabbro samples from the tholeiitic rocks of the South Apuseni Ophiolites yielded Oxfordian ages of 161 ± 4 Ma and 158 ± 1 Ma (Pană et al. 2002a). Based on the age of the ophiolites with the Late Jurassic to Early Cretaceous platform carbonates which overlay the contact between the South Apuseni Ophiolites and the underlying Vidolm Nappe (Sǎsǎran 2005), a late Oxfordian ($\sim$155 Ma) emplacement of the ophiolites can be inferred (Csontos and Vörös 2004; Schmid et al. 2008; Kounov and Schmid 2013). This inferred age is quasi-contemporaneous with the cooling of the Vidolm Nape below the $\sim$550 $^{\circ }{\mathrm{C}}$ isotherm recorded by a $\sim$156 Ma Ar–Ar hornblende age (Dallmeyer et al. 1999), which indicates significant Late Jurassic tectonism at the eastern periphery of the Apuseni Mountains.

#### Early Cretaceous deformation

Deformation during the Early Cretaceous is widely documented to be associated with nappe stacking within the Dacia Mega-Unit (referred to as Austrian Phase in the literature; Sǎndulescu 1984; Schmid et al. 2008; Necea 2010; Gröger et al. 2013; Kounov and Schmid 2013). Eleven Ar–Ar muscovite plateau ages in the 124–100 Ma range (Dallmeyer et al. 1999) provide a record of Alpine tectonism in the basement rocks of the SE Apuseni Mountains (Pană and Erdmer 1994). Based on biostratigraphic evidence, Austrian Phase deformation is constrained to the 136–105 Ma time interval (Kounov and Schmid 2013). Ar–Ar hornblende ages (118 and 119 Ma) from the Baia de Arieş Nappe (Biharia Nappe System) document Early Cretaceous cooling from medium-grade metamorphic conditions (Pană 1998; Dallmeyer et al. 1999). On the basis of geochronological constraints and kinematic analysis (indicators), the direction of thrusting during the Austrian Phase is inferred as E- to NE-facing (present-day coordinates) in the Transylvanian Basin and the East Carpathians (Schmid et al. 2008; Necea 2010; Gröger et al. 2013). Due to Late Cretaceous overprinting under sub-greenschist to greenschist-facies conditions, structures related to the Early Cretaceous deformation are still poorly constrained in the eastern Apuseni Mountains. Syn- to post-tectonic flysch-like sediments which unconformably overlie the tectonic units of the Biharia Nappe System provide additional constraints on this Early Cretaceous deformation (e.g. Bleahu et al. 1981; Suciu-Krausz et al. 2006; Kounov and Schmid 2013).

#### Late Cretaceous deformation

NW-directed thrusting of the Biharia Nappe System on top of the Tisza Mega-Unit is referred to as Turonian Phase (Sǎndulescu 1984; Schmid et al. 2008; Merten et al. 2011; Kounov and Schmid 2013). Kounov and Schmid (2013) interpreted zircon fission-track ages between 95 and 71 Ma from basement units in all but the structurally highest nappe (i.e. Vidolm Nappe) to record erosional and partly extensional denudation following a Turonian, top-NW event. This phase of shortening is geodynamically linked to the N- to NE-directed subduction of the Neotethys (Vardar) ocean beneath Europe-derived units (e.g. Georgiev et al. 2001; Heinrich and Neubauer 2002; Neubauer et al. 2003; Von Quadt et al. 2005). Kinematic indicators and stretching lineation yield evidence for NW-directed thrusting and nappe stacking not only in the Dacia Mega-Unit of the southern and eastern Apuseni Mts. (Balintoni et al. 1996), but also in the Tisza Mega-Unit (Haas and Péró 2004). No evidence of Turonian compressive deformation can be found in the East Carpathians (Culshaw et al. 2012; Gröger et al. 2013). Pană (1998) and Dallmeyer et al. (1999) document complex strain partitioning around the exposed Bihor basement: N- to NW-directed trusting in the western Apuseni Mts. and complex strike slip with a NW thrust component in the eastern Apuseni Mts. is attributed to Early to mid-Cretaceous Ar–Ar ages. Late Cretaceous extension led to the formation of half-grabens and associated hanging-wall deposition of syn- to post-tectonic sediments in the Apuseni Mountains and the South Carpathians (e.g. Lupu and Lupu 1962; Sǎndulescu 1988; Willingshofer et al. 1999; Csontos and Vörös 2004; Schuller 2004; Schuller and Frisch 2006; Schmid et al. 2008; Merten et al. 2011). These predominantly marine sediments represent the erosional products of the previously developed mountain range and seal tectonic nappe contacts of the precedent contractional phase (Balintoni 1994; Schuller 2004; Schuller and Frisch 2006; Schuller et al. 2009). The syn- to post-tectonic deposits are commonly called Gosau-strata in the literature (e.g. Willingshofer et al. 1999; Schuller 2004; Kounov and Schmid 2013). However, since there are similarities as well as differences between the Gosau occurrences in the Apuseni Mountains and the Eastern Alps (see discussion in Schuller et al. 2009), these sedimentary rocks will be further referenced as “Gosau-type”-sediments. The deposition of the “Gosau-type”-sediments during the Late Cretaceous is contemporaneous with magmatic activity along the Apuseni–Banat–Timok–Srednogorie magmatic arc in the Carpathian–Balkan orogen. The ages of calc-alkaline intrusions (“Banatites”; e.g. von Cotta 1865; Berza et al. 1998; Heinrich and Neubauer 2002; Zimmerman et al. 2008) in the Apuseni Mountains range between 81 and 76 Ma (U–Pb in zircon) and are associated with the closure and subduction of the Neotethys ocean (e.g. Von Quadt et al. 2005; Gallhofer et al. 2015).

#### Latest Cretaceous compression

Compressional deformation prevails during the Late Cretaceous–Palaeocene time interval (Merten et al. 2011). This latest Late Cretaceous compression is referred to as “Laramian” Phase in the local literature (Ianovici et al. 1976; Bleahu et al. 1981; Sǎndulescu 1984; Balintoni 1994) and mainly affected the Biharia Nappe System and the adjacent Transylvanian Basin (Huismans et al. 1997; Merten 2011). Based on geometrical relationships of nappe contacts, this deformation phase in the eastern Apuseni Mts. is tentatively associated with collision at the exterior of the Carpathian orocline, i.e. retrovergent deformation associated with thrusting of Ceahlau–Severin Ophiolitic Units over more external units (Schmid et al. 2008; Merten et al. 2011). The collision of the Tisza–Dacia Mega-Unit with the Danubian Block/Moesia in the South (Schmid et al. 1998), the subduction of Neotethys, and the subsequent closure of the Sava-Zone in the West (Ustaszewski et al. 2009) add to the complexity of this latest Late Cretaceous interval. Final uplift and erosion of the Apuseni Mountains also referred to as Bihor doming, occurred during the latest Late Cretaceous–Palaeogene interval (Merten et al. 2011).

## Tectonic units of the study area

### The Biharia Nappe System of the Dacia Mega-Unit

Within the study area, the Biharia Nappe System consists of several thrust sheets that are from bottom to top the Biharia Nappe sensu strictu, the Baia de Arieş Nappe, and the Vidolm Nappe (Fig. 2; Ianovici et al. 1976; Sǎndulescu 1984; Bordea et al. 1988; Pană 1998; Balintoni and Puşte 2002). Penetratively sheared chlorite–phyllonites, variably phyllonitized amphibolites and granitoid rocks, discontinuous lenses of sericite–chlorite–schists, quartzites, and variably dolomitic marbles (Mesozoic cover?) represent the dominant lithologies of the Biharia Nappe System.

#### The Vidolm Nappe and South Apuseni Ophiolites

Pană (1998) introduced the Vidolm Nappe as a separate nappe of the Biharia Nappe System. It is characterized by garnet-, staurolite-, kyanite-, and sillimanite-bearing paragneisses and micaschists representing the structural top of the Biharia Nappe System. Previously, the Vidolm Nappe was treated as a part of the Baia de Arieş Nappe (Kräutner 1980).

In the Southern Apuseni Mts., the Biharia Nappe System is overlain by the South Apuseni Ophiolites which comprise Jurassic MORB-type oceanic lithosphere, Late Jurassic intra-oceanic island arc volcanics, and a Late Jurassic to Cretaceous sedimentary succession (Fig. 2). The South Apuseni Ophiolites are correlated with the Transylvanian Ophiolites (Transylvanides, sensu Sǎndulescu 1984) and assigned to the Eastern Vardar Ophiolitic Unit (Schmid et al. 2008). These ophiolites comprise a Jurassic MORB-type oceanic lithosphere, covered by Late Jurassic, intra-oceanic island arc volcanic products with a thickness of up to 1000 m (Savu et al. 1992; Bortolotti et al. 2002; Pană et al. 2002b; Nicolae and Saccani 2003; Bortolotti et al. 2004). Southwest of Cluj, in the Trascǎu Mountains, Late Jurassic to Early Cretaceous platform carbonates (Oxfordian–Valanghinian) overlie the contact between the South Apuseni Ophiolites and the Vidolm basement (Sǎsǎran 2005). Syn-tectonic Barremian to mid-Albian flysch-like sediments (Feneş-Fm. and Meteş-Fm.; Bleahu et al. 1981; Ellero et al. 2002; Suciu-Krausz et al. 2006; Bălc et al. 2007) transgressively cover the Vidolm Nappe, the South Apuseni Ophiolites, and their carbonate platform cover (Bleahu et al. 1981). The Feneş and Meteş Formations are separated by Aptian microconglomerates and shales which show a change to longer transport distances from the sedimentary source (Valea Dosului Fm.; Bleahu et al. 1981; Suciu-Krausz et al. 2006). The aforementioned Early Cretaceous sediments are unconformably overlain by post-tectonic cover sequences of Late Albian to Cenomanian age (e.g. Valea lui Paul Formation and basal parts of the Rameţi flysch; Ianovici et al. 1976; Bleahu et al. 1981; Bălc et al. 2007; Suciu-Krausz et al. 2006; Kounov and Schmid 2013).

#### Baia de Arieş and Biharia s.str. nappes

**Fig. 3:**
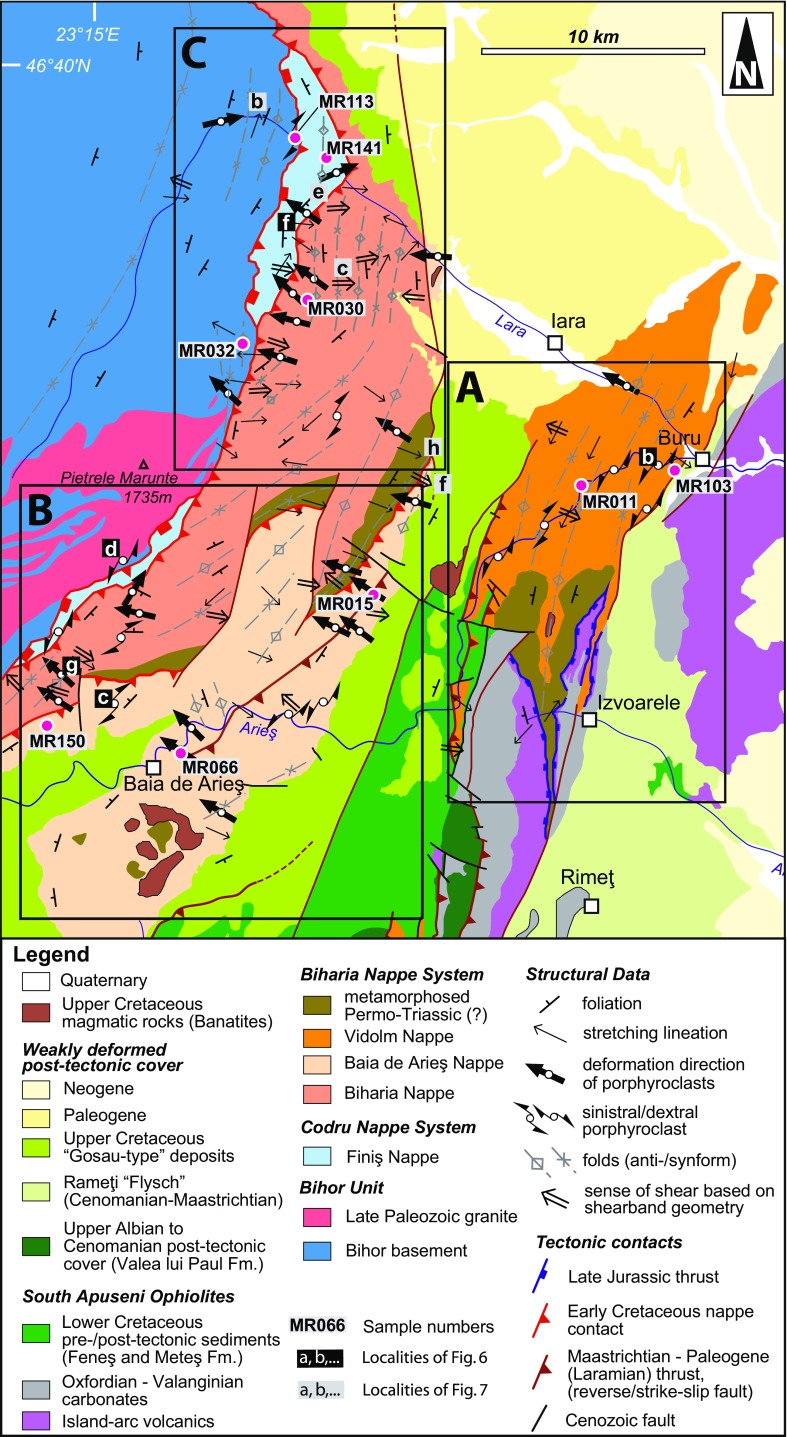
Overview and summary of structural data (original figure modified from Kounov and Schmid 2013). Orientation of main foliation, stretching lineation, kinematic indicators, and fold axes is given on the map. Sample localities of Figs. 6 and 7 (*lowercase letters* according to the picture) and sample names are given in the map. The study area is divided into three sectors (*A*, *B*, *C*) which are referenced in the text and in Fig. 2

Lower sections of the Biharia Nappe System are represented by the Baia de Arieş Nappe and the Biharia Nappe s.str., which are separated by a Mesozoic metasedimentary sequence (Fig. 3). The Biharia Nappe s.str. is characterized by a pervasive greenschist-facies overprint (albite, quartz, sericite, chlorite,  ±carbonate,  ±magnetite and  ±epidote). Phyllonites and metagranites are the dominant lithologies, but amphibolites and lenses of calcitic and dolomitic marbles, often associated with quartzites, characterize the retrogressed portions of pre-Mesozoic basement. The intense chloritization of biotites from the Biharia Nappe s.str. made it impossible to produce biotite separates for Rb–Sr analyses. A polyphase medium-grade metamorphism followed by a retrograde overprint was interpreted for the Baia de Arieş Nappe (Balintoni and Iancu 1986). Garnet, staurolite,  ±kyanite indicate medium-grade metamorphism for the Baia de Arieş Nappe, but the presence of chlorite indicates retrograde overprint. However, the Baia de Arieş Nappe exhibits significantly less retrograde overprinting than the Biharia Nappe sensu strictu (cf. Fig. 5c, d). Furthermore, concordant Ar–Ar hornblende (ca. 118 and 119 Ma) and Ar–Ar muscovite (ca. 117 and 111 Ma) plateau ages suggest rapid cooling following higher-grade penetrative tectonothermal activity of the Baia de Arieş Nappe during the Early Cretaceous (Dallmeyer et al. 1999). Differences between Ar–Ar hornblende ages in the Baia de Arieş (ca. 118 and 119 Ma) and Vidolm (156 Ma) nappes suggest that they were transported to relatively shallower crustal levels at different times (Dallmeyer et al. 1999). Despite the fact that the Biharia and Baia de Arieş nappes differ in the degree of retrograde overprint and their protoliths (Pană and Balintoni 2000), they show similar structural features and are dominated by top-NW-directed thrusting (Fig. 3).

### Tisza Mega-Unit

### Codru basement sliver

The Codru Nappe System tectonically overlies the Bihor Unit. West of the exposed Bihor basement, in the Codru Mts., the nappe stack of the Codru Nappe System predominantly consists of sedimentary rocks (Bleahu et al. 1968). The end of Mesozoic sedimentation on top of the Codru Nappe System is constrained to the Barremian (130–125 Ma; Haas and Péró 2004). Permian quartz-porphyries, associated with sandstone and conglomerates, are present at the base of the Mesozoic succession, but only one nappe (the Finiş-Gîrda Nappe) contains crystalline basement (Bleahu et al. 1981). Although distinctly different and situated in a different mountain range (Pană et al. 2002b), this basement has been assumed (on geometrical criteria) to be correlative to a sliver of amphibolite-dominated crust that frames the Bihor basement to the south and east and has no sedimentary cover (Ianovici et al. 1976; Sǎndulescu 1984). This narrow basement unit crops out in the study area and will be referred to as Codru Nappe (Figs. 1, 2). The lithologies of this basement sliver mainly consist of amphibolites, metagranitoids with subordinate paragneiss, micaschist, and quartzites which were overprinted by anastomising greenschist to sub-greenschist shear zones and faults (Highiş-Biharia and Trascău Shear Zones, sensu Pană and Erdmer 1994; Pană 1998). Pană and Erdmer (1994) report a minimum of 20 km of sinistral displacement along the Highiş-Biharia Shear Zone, which can be traced for more than 200 km along the boundary between the Tisza and Dacia Mega-Units. Basement rocks of the Codru Nappe show amphibolite-facies metamorphic overprint and strong retrogressive chloritization in the study area. Biotite-rich veins, related to the metagranites cross-cutting the amphibolites, were used for Rb–Sr biotite dating.

### Bihor Unit

The Bihor Unit (“Bihor parautochthon”) is the lowest unit in the present-day nappe stack of the Apuseni Mountains and mainly consists of polyphase metamorphic paragneisses, amphibolites, micaschists, and the Permian Muntele Mare granite (Ianovici et al. 1976; Kräutner 1980; Bleahu et al. 1981; Hârtopanu and Hârtopanu 1986; Balintoni et al. 2009). On the west side of the Bihor gneiss–granite basement, a non-metamorphosed Permian–Early Cretaceous sedimentary sequence unconformably overlies these basement rocks (Fig. 1; Ianovici et al. 1976; Bleahu et al. 1981), whereas in the study area, only basement rocks are outcropping. The end of the Mesozoic deposition in the Bihor Unit is constrained by Albian to Turonian strata, which are tectonically overlain by the Codru Nappe System (see 50,000 map sheet Biharia; Bordea et al. 1986). Further west, outcrops of the Bihor Unit can be found in Croatia (Slavonian Mountains) and in the Villany and Papuk Mountains in Hungary (Schmid et al. 2008). Intersecting index mineral zones document two medium-grade metamorphic events in the study area (Hârtopanu and Hârtopanu 1986). Unfortunately, no information on the absolute timing of these medium-grade metamorphic events is available. Ar–Ar muscovite and hornblende data by Dallmeyer et al. (1999) indicate a gradient from Variscan ages in the western, non-retrogressed portion of the Bihor basement to Alpine ages towards its eastern, retrogressed periphery. Greenschist-facies biotite/chlorite zones intersecting the Barrovian isogrades are interpreted to represent a later cycle, which also overprinted the Mesozoic cover of the Bihor Unit (only sub-greenschist-facies) and its neighbouring tectonic units (Pană 1998; Árkai et al. 2000; Árkai 2001; Biševac et al. 2010; Balen et al. 2013).

## Kinematic analysis

**Fig. 4:**
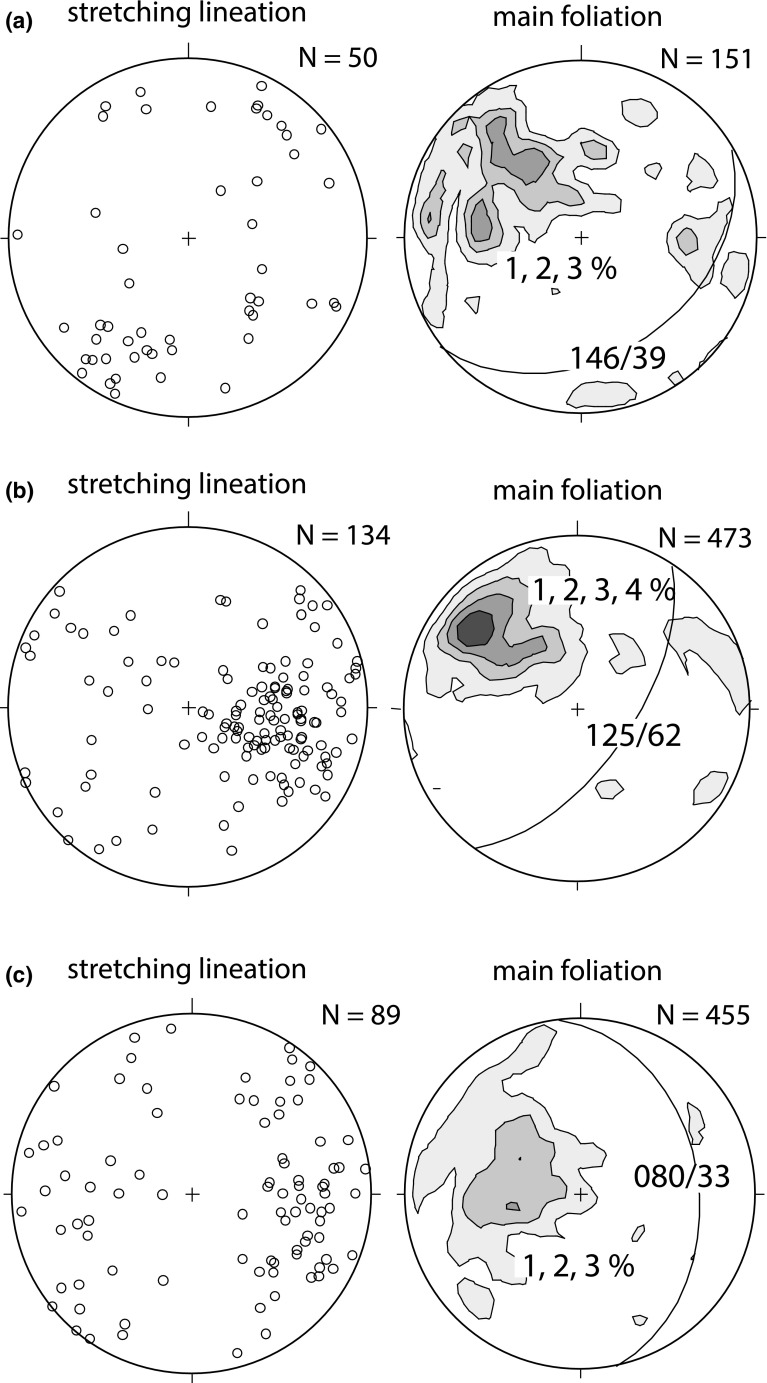
Equal-area, lower-hemisphere stereographic plots of stretching lineation and contoured pi-plots of main foliation, separated according to the sectors given in Figs. 2 and 3: **a** sector A from the Vidolm Nappe shows a dominant NE–SW-trending stretching lineation, strike parallel to the SE-dipping main foliation. East and south-dipping foliation at several sites is due to km-scale open folding; **b** sector B exhibits a dominant NW–SE-trending stretching lineation, parallel to the dip of the main foliation; **c** sector C shows a slightly more E–W-trending direction of stretching lineation and E-dipping main foliation

**Fig. 5:**
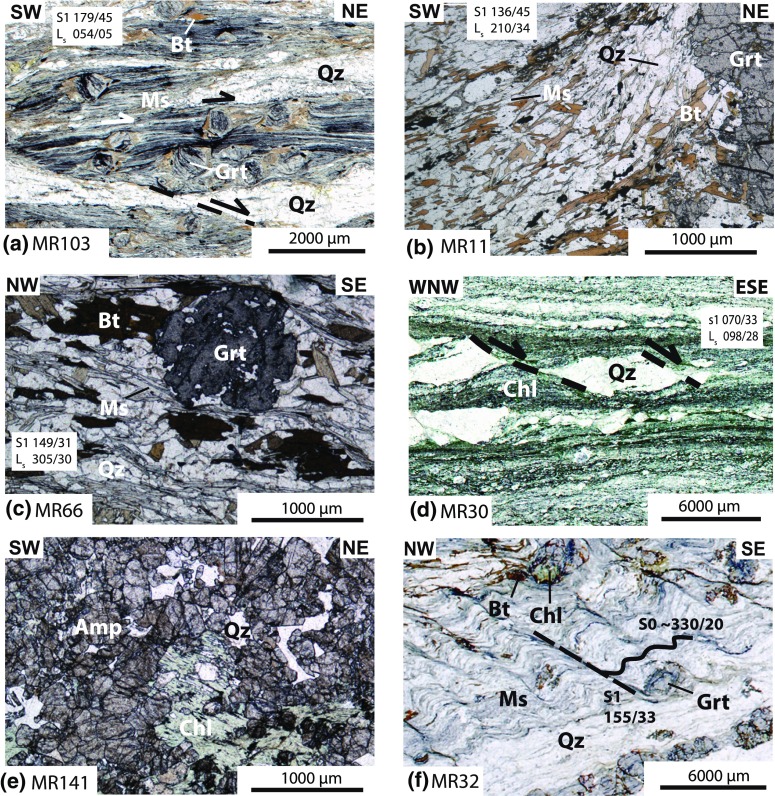
Thin sections of Vidolm, Baia de Arieş, Codru, and Bihor Units, normal to S1 and parallel to L1. Sample localities are indicated in Fig. 3. Abbreviations of mineral names are in accordance with Whitney and Evans (2010): *bt* biotite, *chl* chlorite, *grt* garnet, *ms* muscovite, *qz* quartz, *st* staurolite. **a** Sample MR103 was taken from the eastern periphery of the Vidolm Nappe. The thin section shows a mylonitic fabric and ductile NE-directed shearing of quartz bands. Inclusion-rich garnet sigma-clasts (up to 1.5 mm in diameter) also exhibit top-NE-directed shear senses. Biotites in the pressure shadows of the sigma-clasts indicate syn- to post-kinematic growth. **b** Sample MR11 represents a paragneiss of the central part of the Vidolm Nappe, along the Arieş valley, and was used for Ar–Ar and Rb–Sr analyses. The main foliation consists of non-retrogressed biotite and muscovite and shows only little deformation. Garnets of up to 2 mm in diameter show retrograde metamorphosis into biotite and chlorite. **c** Sample MR66 was prepared from garnet-bearing paragneiss from the central part of the Baia de Arieş Nappe. Large-scale fold axes strike NE–SW, and sigma-clasts in the vicinity of the outcrop indicate top-NW thrusting. The thin section shows an anastomising muscovite foliation bending around zoned garnet minerals (up to 1.7 mm in size). Large, non-retrogressed biotite flakes measuring up to 1 mm on their longest axis show syn- to post-kinematical growth. **d** Strongly retrogressed quartzitic mylonites from the Biharia Nappe s.str. (sample MR30) show boudinage and top-E shearing of quartz clasts. **e** Downstream from the contact of Bihor Unit and Codru Nappe System, a coarse grained (${<}$5 mm) biotite-rich vein within amphibolites of the Codru Nappe System was sampled and analysed (sample MR141). **f** Sample MR32 represents a micaschist from the Bihor Unit. A NE–SW-trending, WNW-vergent crenulation cleavage (s1) overprints an older, NW-dipping foliation (s0). The crenulation cleavage is discrete in the mica-rich layers and less developed in the more quartz-rich layers. Chloritized biotites indicate retrogressive overprinting

**Fig. 6:**
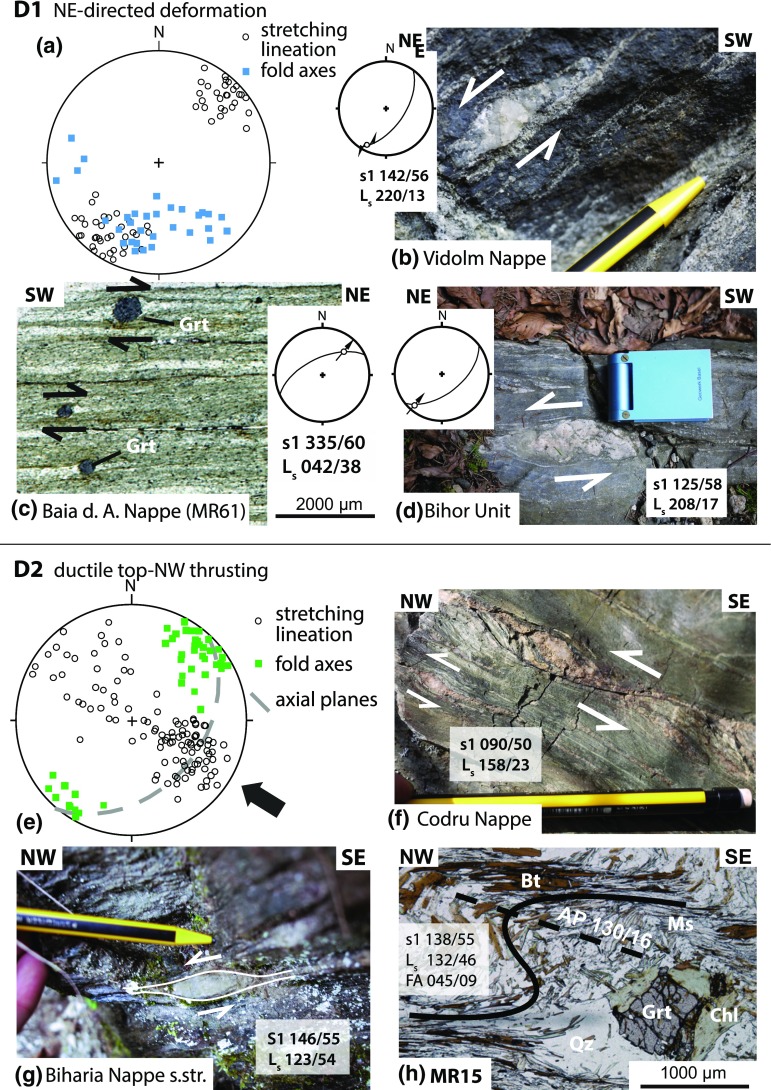
Ductile structures attributed to the deformation phases D1 and D2. D1 summarizes complex, NE-directed deformation as shown through stretching lineation and associated inclined folds (**a**). Intense folding during the subsequent D2 phase is responsible for varying strike-slip (**b**), normal fault (**c**), and thrust geometries (**d**) of the D1-phase. D2 is associated with NW–SE-trending stretching lineation (**e**) and summarizes top-NW thrusting (**f**, **g**), nappe stacking and associated NW-vergent folding (**h**) of the tectonic units in the study area

**Fig. 7:**
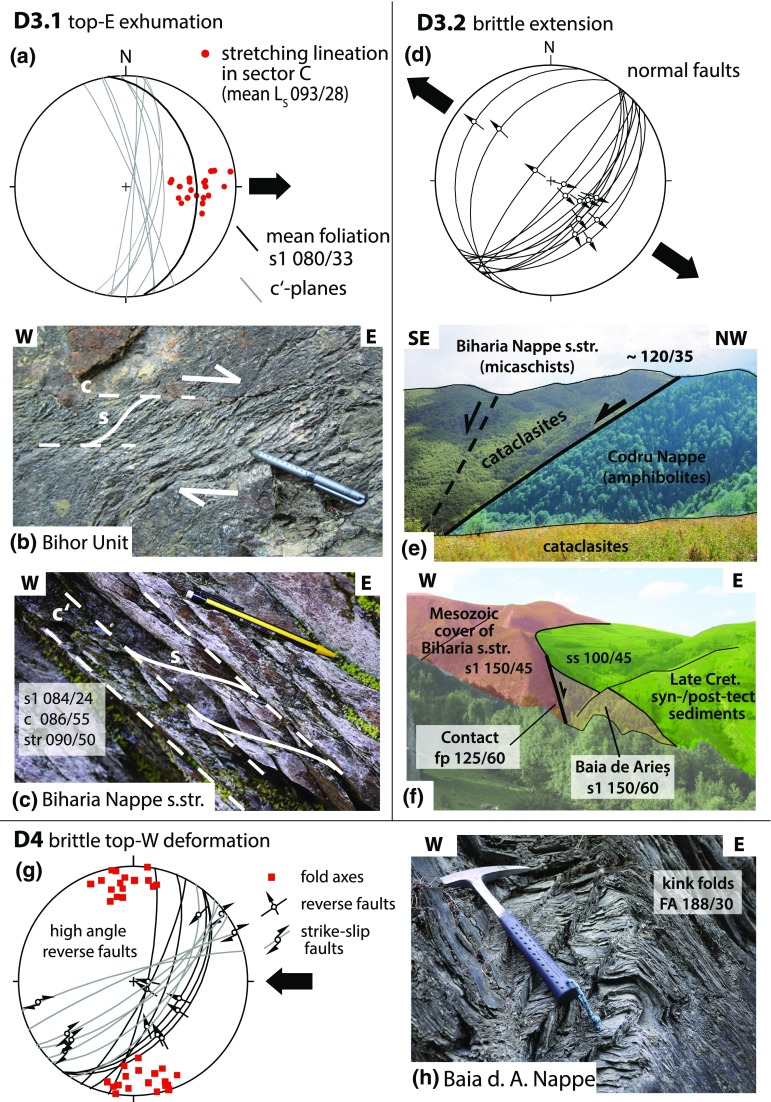
Overview over the structures attributed to the deformation phases D3 and D4. D3.1 represents E-directed ductile shear zones (**a, b**) and shear bands (**c**) associated with the exhumation of the Bihor Unit. D3.2 summarizes a brittle extensional phase that overprints the previously established nappe contacts (**d, e**). This extension is associated with hanging-wall sedimentation of syn- to post-tectonic sediments (**f**). D4 summarizes the brittle, top-W-directed reactivation of the nappe contacts in the study area (**g**), illustrated through kink folds in mylonites of the Baia de Arieş Nappe (**h**)

Field data include measurements of foliation, stretching lineation, kinematic indicators, and fold geometries which are shown on the map in Fig. 3 and in the plots in Fig. 4. Together with petrographical and microstructural analyses (Fig. 5) and available thermochronological data, a relative chronological order was worked out (shown in Figs. 6, 7). No structural data can be clearly assigned to the Late Jurassic obduction of the South Apuseni Ophiolites on top of the Vidolm Nappe. However, since the obduction is the precursor for the Alpine evolution, it will be addressed as D0.

### D1: NE-directed deformation

Horizontal, NE–SW-trending stretching lineation (Fig. 4b) is characterized by quartz and/or feldspar mineral growth oriented parallel to the strike of the SE-dipping main foliation. The lineation trends sub-parallel to the NE–SW-trending fold axis of isoclinal folds and its axial plane foliation. This NE–SW-trending stretching lineation is overprinted by a NW–SE-trending stretching lineation associated with the D2 deformation and thus indicates a previous (i.e. pre-D2) deformation phase.

Garnet and quartz–porphyroclasts indicating dextral and sinistral sense of shear are associated with this strike-parallel lineation; their three-dimensional relationship with the main foliation shows strike-parallel E- to NE-directed movement along sinistral strike-slip, thrust, and normal fault geometries (Fig. 6b–d). The occurrence of both dextral and sinistral sigma-clasts and the variation in kinematic relationships relates to folding of the main foliation during subsequent NW-directed nappe transport (D2). Complex pattern of polyphase folding can be observed in several outcrops. The Vidolm Nappe shows dominant NE–SW-trending, horizontal stretching lineation and sinistral/dextral porphyroclasts which indicate intense shearing during D1 (sector A in Figs. 2, 3, 4a). The oriented thin section of sample MR103 in Fig. 5a shows a mylonitic fabric with shearbands, folded muscovites, strongly deformed quartz bands, and retrogressed garnet–porphyroclasts. Biotite generally grows in the pressure shadow of the garnet–porphyroclasts, but some grains also overgrow the folds. The rotation of garnet–porphyroclasts indicates top-NE-directed deformation.

### D2: ductile top-NW thrusting and nappe stacking

Top-NW thrusting represents the main deformation phase in the study area and is responsible for the structure and geometry of the present-day nappe stack. Phyllonitization of the nappe contacts and a pervasive retrogressive overprint under greenschist-facies conditions is attributed to this thrusting event. The main foliation (s1) shows a dominant SE dip with a NW–SE-trending, down-dip stretching lineation folded around NE–SW-trending, horizontal fold axes with SE-dipping axial planes (Fig. 6e). Associated kinematic indicators suggest top-NW nappe transport during D2 (Figs. 3, 6f, g). The Bihor Unit exhibits a continuously intensifying retrogressive overprint towards the nappe contact with the Codru Nappe: an earlier, pre-Alpine(?) foliation (s0 in Fig. 5f) gets progressively folded and finally erased and replaced by a SE-dipping foliation (s1) close to the nappe contact. Oriented thin sections (sample MR15; Figs. 3, 6h) reveal a folded main foliation which consists of muscovite and biotite. The chloritization of biotite and garnet minerals indicates retrogressive overprint (sample MR15, Fig. 6). NW-vergent micro- and macrofolds and garnet–porphyroclasts indicate top-NW thrusting. Locally, the Baia de Arieş Nappe shows signicantly less deformation and only a weak retrogression. In these parts, biotite and garnet are well preserved (e.g. sample MR66, Fig. 5c).

### D3.1: top-E exhumation

Kinematic data from the Bihor Unit in the northern part of the study area (sector C in Figs. 2, 3) show evidence for E-directed exhumation of the Bihor Unit in the northern part of the study area (sector C). E-directed shear zones (Fig. 7a, b), a dominantly E-dipping main foliation and E–W to ESE–WNW-trending, down-dip lineations (Fig. 4c), indicate E-directed movement of the hanging wall. Shear bands in the Biharia Nappe s.str. (Fig. 7c) indicate E-directed kinematics and disagree with top-NW-directed movement during D2, as reflected by shear bands in other parts of the study area (e.g. sector B). Since shear bands indicate kinematics at the ductile–brittle transition, E-directed movement under cooler conditions than during the retrogressive overprint (D2) can be inferred.

### D3.2: brittle extension

Normal faults in the study area exhibit a dominant NE–SW strike and top-SE extension (Fig. 7d). The brittle normal faults overprint ductile detachments and are responsible for intense cataclastic reworking of nappe contacts (Fig. 7e). Hanging-wall sedimentation of Late Cretaceous, syn- to post-tectonic sediments subsequently seals the extensional contact between the Baia de Arieş and Biharia s.str. (Fig. 7f).

### D4: brittle E–W compression

Characteristic structures of this tectonic phase in the Apuseni Mountains and adjacent areas are top-NW to top-W reverse faults (Puini and Mezeş thrusts), strike-slip deformation, and N–S-trending open folds (Fig. 7g, h) (Balintoni 1994; Krézsek and Bally 2006; Schuller and Frisch 2006; Merten et al. 2011). Late Cretaceous sediments are tilted, folded, and overthrust by crystalline basement and Early Cretaceous sediments (Fig. 3). N–S-trending, open folds with upright axial planes affect both the crystalline basement and their Early- to Late Cretaceous sedimentary cover. NE–SW-trending D2 fold axes appear to bend into a N–S direction towards the northern part of the study area (sector C in Fig. 3).

## Geochronology

The Ar–Ar muscovite and Rb–Sr biotite methods were chosen to provide age data for a temperature range from $\sim$450 to $\sim$300 $^{\circ }\mathrm {C}$. Extensive fieldwork in the south-eastern part of the Apuseni Mountains (Trascǎu Mts.) and structural and petrographic analyses were carried out to carefully select sample locations that complement and extend already available geochronological data. Where the samples were suitable, biotite and muscovite concentrates were prepared from the same sample. Mechanical mineral separation for Ar–Ar and Rb–Sr isotope analyses were carried out at the Institute of Geology at the University of Innsbruck and at the Geological Survey of Austria in Vienna. Samples of up to 5 kg were processed by conventional mineral separation techniques to prepare biotite and muscovite separates. Weathered surfaces were removed from the sample material before starting the separation procedure. After crushing, grinding, and sieving (${<}$315 $\upmu$m) of the rock samples, the minerals were concentrated with standard separation techniques. Handpicking under an optical microscope was the final stage for muscovite separation in order to provide pure mineral concentrates.

### Methods

#### Rb–Sr in biotite

Biotites were separated from sieve fraction 0.2–0.3 mm using a vibration table, grinding in alcohol and magnetic splitting. The weights of the samples used for dissolution were about 100 mg for whole-rock powder and $\sim$200 mg for biotite. Chemical preparation was performed at the Geological Survey of Austria in Vienna and at the Department of Lithospheric Research at the University of Vienna. The chemical sample preparation follows the procedure described by Sölva et al. (2005). Isotope measurements were taken at the Department of Lithospheric Research at the University of Vienna. Spiked Rb ratios were measured at a Finnigan^®^MAT 262, from a Ta single filament, whereas spiked Sr ratios were analysed at a ThermoFinnigan^®^Triton TI TIMS and run from Re double filaments. During the periods of measurements, the SRM NBS-987 yielded a ratio of $^{86}\mathrm{Sr}/^{87}\mathrm{Sr}$ = 0.710235 ± 5 2$\sigma _m$ (n =7 , December 2010) and 0.710276 ± 3 2$\sigma _m$ (*n* = 12, November 2011–April 2012), respectively. Calculation of ages was done using Isoplot software Version 4.16 (Ludwig 2008) assuming an uncertainty of 1 % on the $^{87}{\rm Rb}/^{86}{\rm Sr}$ ratios. Uncertainties on the $^{87}\mathrm{Sr}/^{86}\mathrm{Sr}$ isotope ratios are quoted as 2$\sigma _m$. Biotite ages were calculated using the Rb-decay constant of 1.393 ± 0.004 $\times \,10^{-11}\,{\rm year}^{-1}$ proposed by Nebel et al. (2011). All uncertainties and final ages are quoted at the 2$\sigma$ level.

#### $^{40}$Ar/$^{39}$Ar muscovite

Isotopic dating of muscovite was carried out in the $^{40}\mathrm{Ar}/^{39}\mathrm{Ar}$ laboratory of the Department of Mineralogy, University of Geneva, Switzerland. The samples were measured with an Argus (GV Instruments) multicollector mass spectrometer, equipped with four high-gain (10$^{12}$ Ohms) Faraday collectors for the analysis of $^{39}\mathrm{Ar}$, $^{38}\mathrm{Ar}$, $^{37}\mathrm{Ar}$, and $^{36}\mathrm{Ar}$, as well as a single Faraday collector (10$^{11}$ Ohms) for the analysis of $^{40}\mathrm{Ar}$. The automated UHV stainless steel gas extraction line incorporates one SAES AP10 getter and one water-cooled SAES GP50-ST101 getter. Single grains of muscovite (≤0.5 cm across) were step-heated using a defocused 55W Photon Machines IR-$\mathrm {CO_2}$ laser that was rastered over the samples to evenly heat the grains. Samples were measured on the Faraday collectors, and time-zero regressions were fitted to data collected from 12 cycles. Peak heights and blanks were corrected for mass discrimination, isotopic decay of $^{39}\mathrm{Ar}$ and $^{37}\mathrm{Ar}$ and interfering nucleogenic Ca-, K-, and Cl-derived isotopes. The data are baseline-corrected, and error calculations include the errors on mass discrimination measurement and the *J* value. $^{40}\mathrm{Ar}$, $^{39}\mathrm{Ar}$, $^{38}\mathrm{Ar}$, $^{37}\mathrm{Ar}$ and $^{36}\mathrm{Ar}$ blanks were calculated before every new sample and after every three heating steps. $^{40}\mathrm{Ar}$ blanks were between 6.5$^{-16}$ and 1.0$^{-15}$ moles. Blank values for m/e 39 to 36 were all less than 6.5$^{-17}$ moles. Age plateaus were determined using the criteria of Dalrymple and Lanphere (1974), and data reduction utilized ArArCalc (Koppers 2002). The samples were irradiated for 11 h (1 MW) in the Ohio State University, CLICIT facility, and *J* values were calculated via the irradiation of Fish Canyon Tuff sanidines (assuming an age of 28.3 Ma; Renne et al. 2010), which were separated by distances of <1 cm, throughout the columnar irradiation package.

### Results

#### Rb–Sr in biotite

**Fig. 8:**
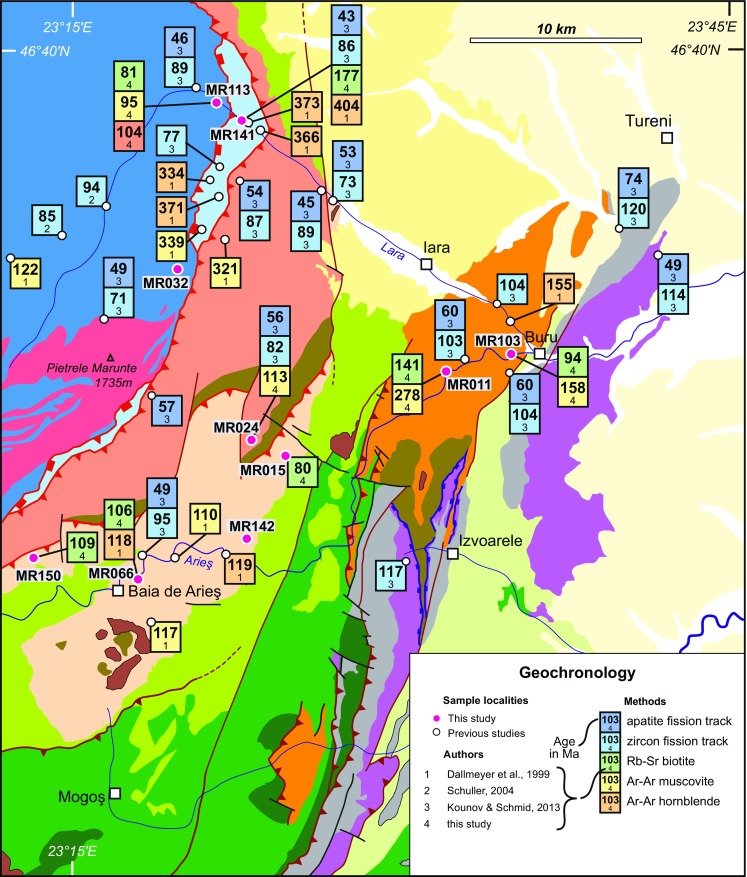
Geological sketch map of the eastern Apuseni Mountains showing existing age dating sample locations and analytical results (modified from Kounov and Schmid 2013). *White dots* are compiled data from Dallmeyer et al. (1999), Schuller (2004), and Kounov and Schmid (2013). *Red dots* are new data presented in this study. Legend is given in Fig. 3

**Fig. 9:**
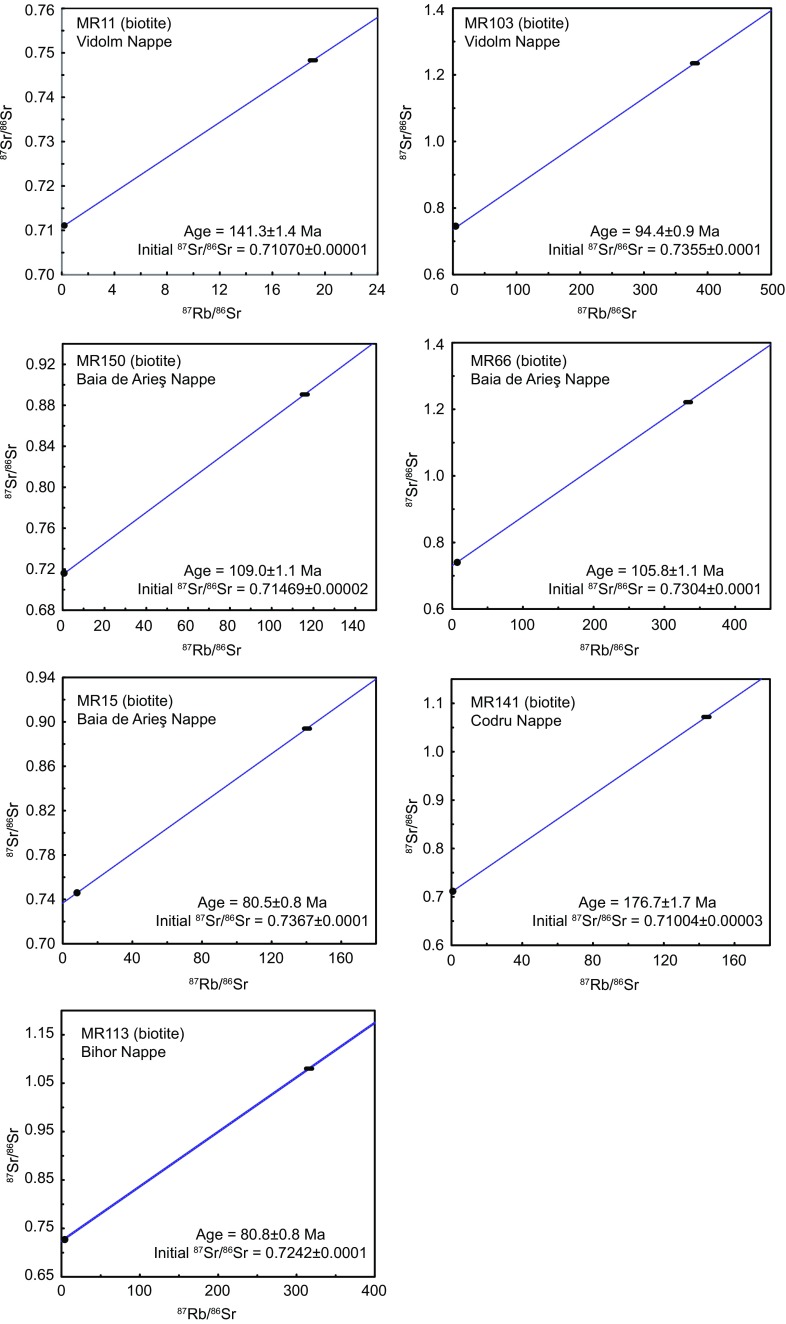
Rb–Sr biotite ages calculated with feldspar (sample MR11) or whole-rock isotope composition (all other samples). Additional data are given in Table 2 in ESM Appendix

Seven biotite concentrates were used for Rb–Sr analyses (see Table 1). Additional data are given in Table 2 in ESM Appendix. With the exception of the Biharia Nappe s.str., all major tectonic units (Bihor, Codru, Baia de Arieş, and Vidolm) were sampled. The high degree of retrogressive metamorphic overprint and the lack of suitable biotite are the reasons for the data gap in the Biharia Nappe. In general, the Rb–Sr biotite data set yields younger ages than corresponding Ar–Ar muscovite ages (Fig. 8; Table 2 in ESM Appendix) and older ages than zircon fission-track data which allows interpreting most Rb–Sr dates as cooling ages. High Rb/Sr ratios, which evidences internal homogeneous and clean biotite separates, are characteristic for all biotite analyses except sample MR11 (141 ± 1 Ma). However, the combination with other thermochronological data (Ar–Ar muscovite and zircon fission-track data) and only little deformation observed in the thin section (Fig. 5b) supports the interpretation of sample MR11 as a cooling age. A Rb–Sr biotite date of the Codru Nappe (177 ± 2 Ma; sample MR141) is significantly older than other Rb–Sr biotite data in the surrounding Bihor and Biharia nappes. Being neither Variscan nor Alpine, this Rb–Sr date can either be interpreted as a mixed age, or as related to a Jurassic tectonic event (e.g. Lower–Middle Jurassic extensional tectonics). This age range is also shown in detrital zircons (population 3: 200–170 Ma) from “Gosau-type” sediments from the study area (Schuller 2004). We cannot provide a conclusive answer for this; however, this date allows constraining the Alpine thermal overprint to less than the closure temperature of the Rb–Sr system in biotite (320 ± 40 $^{\circ }\mathrm {C}$; Harrison and McDougall 1980). Samples MR150 and MR66, both from the southern part of the Baia de Arieş Nappe, yield similar ages (106 ± 1 vs. 109 ± 1 Ma), which show a good agreement with other thermochronological data from the vicinity. Tectonic disturbance of the Rb–Sr system is indicated by the fact that the Rb–Sr biotite date of sample MR103 (94 ± 1 Ma) is younger than a nearby zircon fission-track age (104 ± 6 Ma; Kounov and Schmid 2013). Intense ductile deformation is visible in the thin section (Fig. 5a), and the sample is located in a peripheral area of the Vidolm Nappe. The youngest Rb–Sr cooling ages (80 ± 1 and 81 ± 1 Ma) were obtained from the Baia de Arieş Nappe (sample MR15; Fig. 6h) and the Bihor Unit (sample MR113; Fig. 5e). Both samples show ductile deformation and retrogressive overprint and thus could provide arguments for a tectonic influence on these ages. However, thermal modelling of fission-track data from structurally comparable locations in both units indicates cooling during that time interval (80–60 Ma; Kounov and Schmid 2013) (Fig. 9).

#### $^{40}$Ar/$^{39}$Ar muscovite

**Fig. 10:**
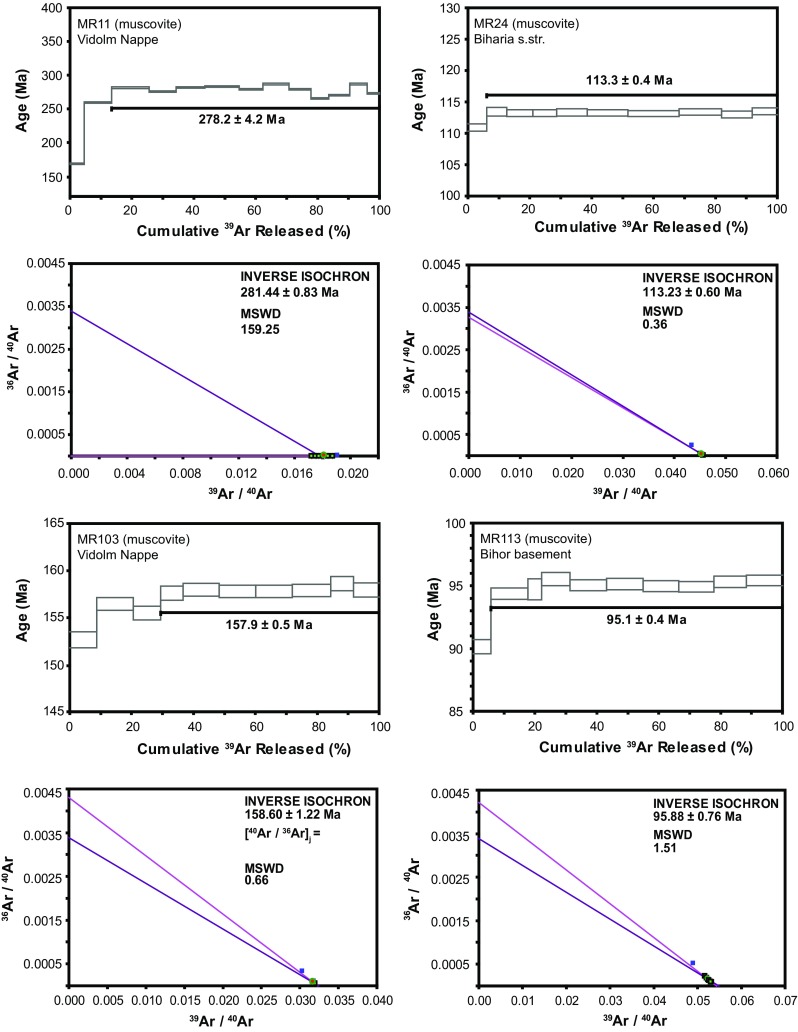
$^{40}\mathrm{Ar}/^{39}\mathrm{Ar}$ age spectra and inverse isotope correlation diagrams for multigrain muscovite concentrates. Sample names are given on the plots. Analytical uncertainties (2$\sigma$, intralaboratory) are represented by *vertical width of bars*. *J* value = 0.0029690 ± 0.0000048. Experimental temperatures increase from *left* to *right*. Ages given on the plot represent plateau spectra. Sample MR011 yields a discordant age spectrum. Heating steps *highlighted in bold* are used to calculate the plateau age for samples MR24, MR103, and MR113. Mass discrimination = 0.9907 ± 0.00334. Data are corrected for blanks, interfering nucleogenic reactions and decay of $^{37}$Ar and $^{39}$Ar. Details of the analytical procedures are given in Table 3 in ESM Appendix

Four $^{40}\mathrm{Ar}/^{39}\mathrm{Ar}$ muscovite samples were prepared from the Bihor (sample MR113), Biharia (sample MR24), and Vidolm (samples MR11 and MR103) nappes to complement the data set of Dallmeyer et al. (1999). Details of the analytical procedures are given in Table 3 in ESM Appendix. All four Ar–Ar muscovite age spectra show a staircase pattern (Fig. 10), some stairs are well expressed reflecting a two-stage tectonothermal evolution, e.g. sample MR11. Sample MR11 does not show a plateau, but that portion is scattered yielding a mean age, probably due to incomplete equilibration of the isotope system. Since the $^{40}\mathrm{Ar}/^{36}\mathrm{Ar}$ intercept on the inverse isochron does not yield a meaningful value, the age of sample MR11 is not a cooling age and has to be interpreted carefully. However, considering the timescale, the difference between the date derived from the age spectra and the date obtained from the inverse isochron (278 ± 4 vs. 281 ± 1) is insignificant. The age spectra dates of samples MR103, MR24, and MR113 match the inverse isochron ages and yield acceptable MSWD values lower than 2.5. The $^{40}\mathrm{Ar}/^{36}\mathrm{Ar}$ intercepts on the inverse isochron of sample MR24 show a good overlap with the atmospheric composition (295.5) and thus confirm an Early Cretaceous cooling age for the Biharia Nappe. Our 113 Ma plateau age from Biharia s.str. (sample MR24) is consistent with Ar–Ar muscovite data from the Baia de Arieş Nappe (Dallmeyer et al. 1999) and strongly suggests that the time of rejuvenation in the Biharia Nappe System is due to Early Cretaceous deformation. Sample MR103 (158 ± 1 Ma) from the Vidolm Nappe correlates well with the $\sim$156 Ma Ar–Ar hornblende plateau age reported by Dallmeyer et al. (1999) from the same unit just 5 km to the north. Our muscovite plateau age of 95 ± 1 Ma (sample MR113) from the eastern periphery of the Bihor unit near the contact with the Codru Nappe corresponds to the plateau age of $\sim$101 Ma reported by Dallmeyer et al. (1999) from an identical structural position in the footwall of the tectonic contact with the Codru Nappe about 10 km farther north.

## Discussion

### The thermal evolution of tectonic units

**Fig. 11:**
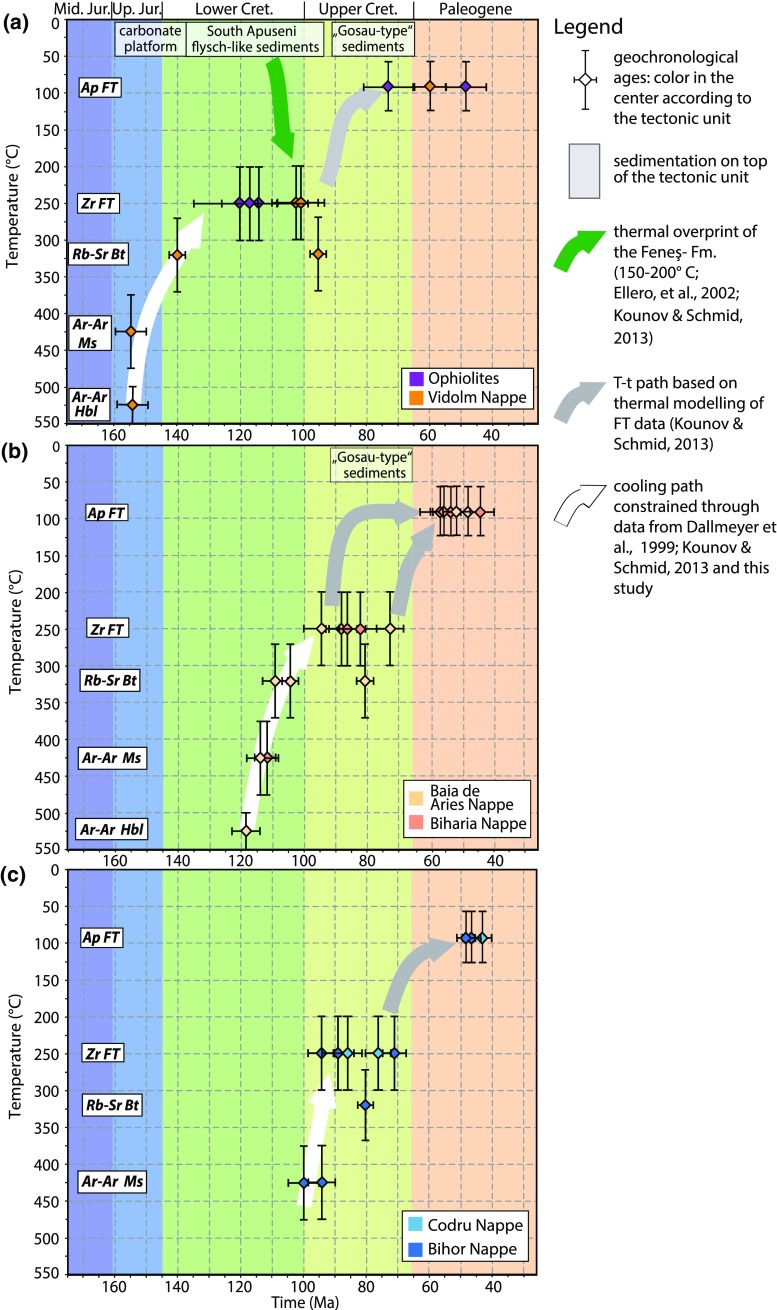
Geochronological data from previous studies (Dallmeyer et al. 1999; Kounov and Schmid 2013) in combination with new data from this study are integrated into time–temperature diagrams for individual tectonic units to illustrate differential cooling from medium-grade (>500 °C) to low-grade thermal conditions (200–300 °C). *Diagrams* are arranged relative to the structural position of the tectonic units: **a** South Apuseni Ophiolites/Vidolm Nappe; **b** Biharia/Baia de Arieş Nappes; **c** Bihor/Codru. Geochronological constraints are the same as in Fig. 8; for further details, see “The thermal evolution of tectonic units” section. The *green arrow* refers to the Early Cretaceous thermal overprint (150–200 $^{\circ }\mathrm {C}$) of the Feneş-Fm. (Ellero et al. 2002; Kounov and Schmid 2013). A *grey arrow* illustrates the cooling path of the tectonic units based on thermal modelling of apatite fission-track data (Kounov and Schmid 2013). A *white arrow* illustrates Early Cretaceous cooling from medium-grade conditions based on thermochronological data from Dallmeyer et al. (1999) and the data presented in this study

New and previously published data sets are integrated into time–temperature paths (Fig. 11). For the interpretation of the geochronological data, closure temperatures of 320 ± 40 $^{\circ }\mathrm {C}$ for Rb–Sr system in biotite (Harrison and McDougall 1980) and 425 $^{\circ }\mathrm {C}$ (Harrison et al. 2009) for the Ar–Ar system in muscovite were used.

#### Vidolm Nappe

The oldest age for the Vidolm Nappe comes from sample MR11 (calculated plateau age: 278 ± 4 Ma). However, it shows a discordant age spectrum and is significantly older than other Alpine ages from the same unit. This indicates a possible internal thermal gradient for the Vidolm Nappe. In combination with the Rb–Sr biotite date from the same sample (141 ± 1 Ma; MR11), we conclude that temperatures in this part of the Vidolm Nappe were sufficient to reset the Rb–Sr system in biotite (${>}$320 ± 40 $^{\circ }\mathrm {C}$), but not high enough to completely reset the K-Ar system in muscovite (${<}$425 $^{\circ }\mathrm {C}$). The thin section of sample MR11 (Fig. 5b) shows barely any retrogression or tectonic disturbance and seemingly escaped major Alpine overprinting. However, other sites from the Vidolm Nappe experienced substantial tectonic and thermal overprint during the Alpine evolution (age data from sample MR103, see also Fig. 5a). The 156 Ma Ar–Ar hornblende age (Dallmeyer et al. 1999) indicates thermal conditions of $\sim$500–550 $^{\circ }\mathrm {C}$ (Harrison and McDougall 1980) in Late Jurassic times followed by slow cooling, probably already reaching surface conditions during the Early Cretaceous (Fig. 11a). Fission-track data from the South Apuseni Ophiolites indicate cooling below the zircon partial annealing zone (200–300 $^{\circ }\mathrm {C}$; Tagami and OSullivan 2005) at around 120 Ma (Kounov and Schmid 2013). Early Cretaceous, syn-tectonic sediments which unconformably overlie the Vidolm Nappe and the South Apuseni Ophiolites constrain a position close to the surface during the Early Cretaceous (Figs. 1, 3). Low-grade metamorphic overprint and deformation of the Feneş-Fm. indicates involvement in Early Cretaceous deformation (Ellero et al. 2002; Kounov and Schmid 2013). The 94 ± 1 Ma Rb–Sr biotite age of sample MR103, situated at the periphery of the Vidolm Nappe, is related to Late Cretaceous (extensional?) overprinting within local shear zones after the uplift above the 320 $^{\circ }\mathrm {C}$ isotherm. However, the lack of Late Cretaceous zircon fission-track ages limits the thermal imprint of Late Cretaceous tectonic events on the Vidolm Nappe to less than the zircon partial annealing zone. Cooling down to surface conditions during Late Cretaceous times is indicated by thermal modelling of fission-track data (Kounov and Schmid 2013) and further constrained by the presence of “Gosau-type” sediments on top of metamorphic basement (Schuller 2004; Fig. 11).

#### Biharia s.str. and Baia de Arieş nappes

The thermochronological data set of the Baia de Arieş Nappe indicates well-constrained, rapid, post-metamorphic cooling from medium-grade (${>}$500 $^{\circ }\mathrm {C}$) to low-grade thermal conditions (200–300 $^{\circ }\mathrm {C}$) during the Late Jurassic–Early Cretaceous (Pană 1998; Dallmeyer et al. 1999; Kounov and Schmid 2013, and this study). Ar–Ar hornblende ages (Dallmeyer et al. 1999) constrain cooling of the rocks below the 550 $^{\circ }\mathrm {C}$ isotherm (Harrison and McDougall 1980) at $\sim$120 Ma. Due to comparable thermochronological ages and the neighbouring position of the Biharia and Baia de Arieş nappes, a common tectonic evolution is inferred (Fig. 11b). However, due to the lack of index minerals, no constraint for thermal peak conditions in the Biharia Nappe s.str. during the Alpine evolution can be given. Based on the intense greenschist-facies overprint of the Biharia Nappe s.str. and Early Cretaceous Ar–Ar muscovite ages (sample MR24; Table 1), we estimate Alpine peak conditions of $\le$450 $^{\circ }\mathrm {C}$. Thermal modelling of fission-track data indicates cooling to the apatite partial annealing zone (60–120 $^{\circ }\mathrm {C}$; Green et al. 1986; Gallagher et al. 1998) during Early–Late Cretaceous times (around 90 Ma; Merten et al. 2011; Kounov and Schmid 2013). The presence of Late Cretaceous “Gosau-type” sediments on top of the Vidolm, Baia de Arieş, and Biharia s.str. Nappes indicates at least partial surface exposure of the Biharia Nappe System during Late Cretaceous times. Late Cretaceous Rb–Sr biotite (80 ± 1 Ma; sample MR15) and zircon fission-track ages (73 ± 4 Ma; Kounov and Schmid 2013) from the eastern periphery of the Baia de Arieş Nappe show slightly delayed cooling with respect to south-western parts. This could relate to differential exhumation of the Baia de Arieş Nappe during Late Cretaceous times. However, thermal resetting by nearby Late Cretaceous intrusions (“Banatites”; Fig. 8) is also possible. By the end of the Late Cretaceous, the basement units had cooled to surface conditions and experienced only very little thermal overprint during the Palaeogene (Merten et al. 2011).

#### Bihor Unit and Codru Nappe

Time–temperature paths of the Bihor Unit show cooling from medium- to low-grade thermal conditions at the transition between Early and Late Cretaceous (Fig. 11c). Ar–Ar data from this study and Dallmeyer et al. (1999) constrain rapid cooling during this time interval. Compared with the other units in the study area, this cooling is delayed and about 20 Ma later than cooling in the neighbouring Dacia Mega-Unit. Age data from sample MR113 (95 Ma Ar–Ar muscovite and 80 ± 1 Ma Rb–Sr biotite), together with zircon fission-track ages, constrain rapid post-tectonic cooling following a Late–Early Cretaceous thermal overprint of peripheral areas. Within the basement sliver of the Codru Nappe, a non-reset Rb–Sr biotite cooling age (MR141), as well as still preserved Variscan Ar–Ar muscovite and hornblende data (Dallmeyer et al. 1999), constrains the temperatures during Alpine deformation to less than the closure temperature of the Rb–Sr biotite system (320 $^{\circ }\mathrm {C}$). On the other hand, temperatures were sufficiently high to produce a Late Cretaceous zircon fission-track age (86 ± 4 Ma; Kounov and Schmid 2013), leaving only a narrow gap between $\sim$200–320 $^{\circ }\mathrm {C}$ (partial annealing zone of zircon fission tracks and $T_c$ of biotite) for the actual thermal conditions in the Codru basement sliver during the Cretaceous. The thermal difference between the basement sliver of the Codru Nappe (*T* < 320 $^{\circ }\mathrm {C}$) and the adjacent Bihor Unit (*T* > 425 $^{\circ }\mathrm {C}$) presumably relates to their different structural position during the Early Cretaceous. Thermal modelling of apatite fission-track data from the Bihor and Codru Units constrains gradual cooling to the apatite partial annealing zone (60–120 $^{\circ }\mathrm {C}$) and shows converging thermal conditions between Bihor and Codru during the Late Cretaceous (Fig. 11c).

### Integrating kinematics and thermochronological data

**Fig. 12:**
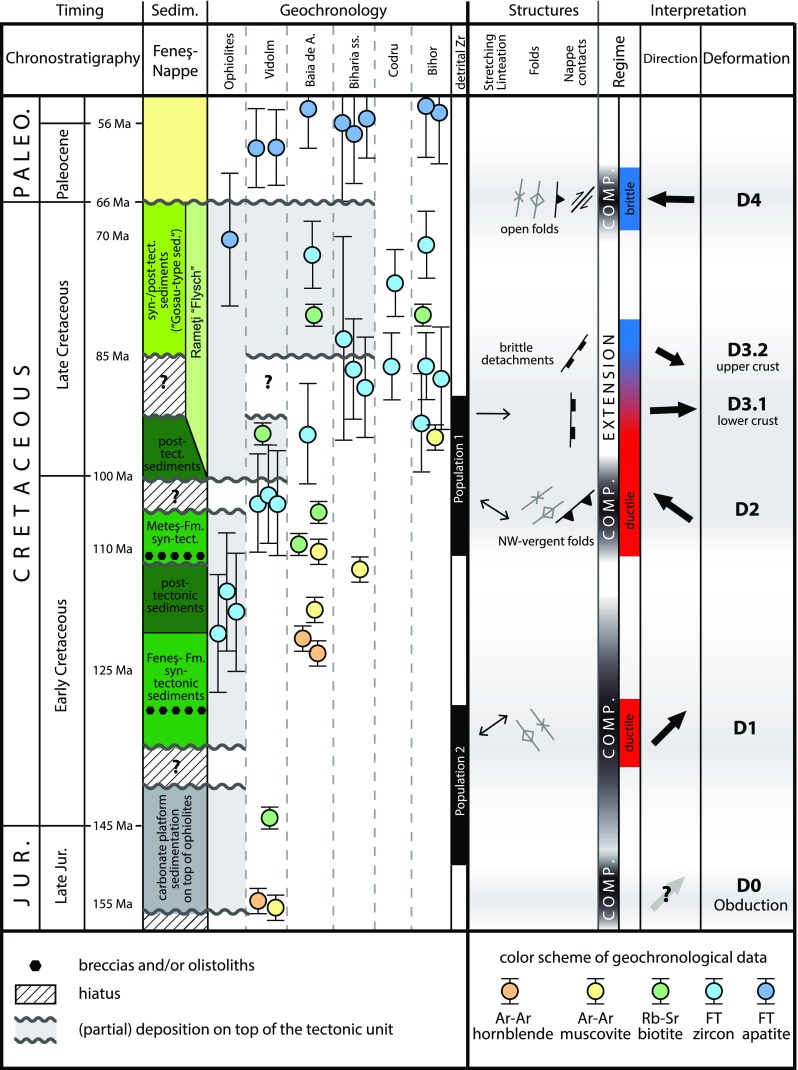
Summary of the results of the present study. Thermochronological (Dallmeyer et al. 1999; Schuller 2004; Kounov and Schmid 2013, and this study), sedimentary (Feneş Nappe Bleahu et al. 1981; Suciu-Krausz et al. 2006), and kinematic data (this study) are integrated and interpreted in the scheme of deformational phases as shown in Figs. 6 and 7

Polyphase overprinting of pre-Alpine basement units and a significant thermal overprint during the Alpine evolution led to a complex structure with strong lateral and vertical gradients. Thus, in the following section, we correlate sedimentary, structural, and thermochronological data to constrain the Alpine tectonothermal evolution of the tectonic units in the study area (Fig. 12).

#### D0: Late Jurassic obduction

The Late Jurassic Ar–Ar hornblende age (155 Ma; Dallmeyer et al. 1999) of the Vidolm Nappe correlates with the emplacement of ophiolitic material on the continental margin (e.g. Csontos and Vörös 2004; Schmid et al. 2008; Kounov and Schmid 2013). Slow cooling of the Vidolm Nappe and the coeval cessation of deposition of platform carbonates on top of the ophiolites (shallowing upward cycles; cf. Sǎsǎran 2005) indicate tectonic uplift of the Vidolm Nappe and the overlying South Apuseni Ophiolites during the Early Cretaceous. In agreement with Kounov and Schmid (2013), early cooling down to temperatures lower than 200 $^{\circ }\mathrm {C}$ around mid-Cretaceous times (Fig. 11a) and the lack of major thermal and structural overprinting afterwards support positioning the Vidolm Nappe on top of the Biharia Nappe System from mid- to Early Cretaceous times onwards. The more or less continuous syn-tectonic sedimentation of Early Cretaceous, flysch-like sediments on southern parts of the Vidolm Nappe (Lupu 1983; Ellero et al. 2002; Suciu-Krausz et al. 2006) indicates at least partial surface exposure of the crystalline basement and the erosion of overlying ophiolites starting with the sedimentation of the Feneş-Fm. around 130 Ma (Fig. 12).

#### D1: Early Cretaceous Deformation

The lack of stratigraphic control and a strong retrogressive overprint during the following tectonic phases complicate constraining Early Cretaceous deformation in the Tisza Mega-Unit. Within the boundaries of the study area, the Codru Nappe did not experience thermal overprint higher than $\sim$320 $^{\circ }\mathrm {C}$ during the Alpine evolution. Stratigraphic and structural evidence from the Codru Mountains, west of the study area, constrains Early Cretaceous top-NE-directed emplacement of the Codru Nappe on top of the Bihor Unit (Sǎndulescu 1984; Haas and Péró 2004; Balintoni et al. 1996). However, the absence of metamorphic overprint in the Western part of the Bihor Unit precludes significant overthrusting. Since the Codru Unit in the study area only experienced low-grade metamorphic overprint during the Alpine evolution, we suggest lateral, orogen-parallel translation to explain its present-day position. Sinistral strike-slip movements between the Tisza and Dacia Mega-Units (Highiş-Biharia shear zone sensu Pană 1998; Dallmeyer et al. 1999) allow to account for oblique compression as recorded by strike-parallel kinematic indicators (Figs. 3, 4). The concordance of Ar–Ar hornblende and Ar–Ar muscovite ages from the Baia de Arieş Nappe (Fig. 11b) is interpreted as rapid cooling following higher-grade penetrative tectonothermal activity during Early Cretaceous times (cf. Dallmeyer et al. 1999). Together with the onset of sedimentation in the Feneş-Fm. ($\sim$130 Ma), this correlates with the timing of top-NE-directed nappe stacking in the Dacia Mega-Unit from  ±135 Ma onwards (Necea 2010; Culshaw et al. 2012; Gröger et al. 2013). The structural data set from the study area (Figs. 3, 4) shows complex geometries for the D1-phase, which presumably are a result of subsequent overprinting during D2. However, the strike-slip, thrust and normal fault geometries share a general, top-NE direction. Thus, we interpret this D1 deformation phase in the study area as an interference between the neighbouring position of the Tisza and Dacia Mega-Units (cf. Schmid et al. 2008; Vissers et al. 2013, Fig. 7) and Early Cretaceous top-NE deformation in internal parts of the Dacia Mega-Unit.

The Feneş-Fm. (130–113 Ma; Bleahu et al. 1981) records the erosional products of the ophiolites and their Mesozoic cover sequence (Suciu-Krausz et al. 2006). A low-grade metamorphic overprint ($\sim$200 $^{\circ }\mathrm {C}$) between 120 and 100 Ma indicates involvement of the Feneş-Fm. in Late–Early Cretaceous orogeny (Ellero et al. 2002; Kounov and Schmid 2013). Changes in the mineralogical content, indicating longer periods of alteration/transportation (Suciu-Krausz et al. 2006) and the absence of breccias in the upper part of the Feneş-Fm., are interpreted as a change in the tectonic regime during the Aptian (i.e. a transition from D1 to D2).

#### D2: Late–Early Cretaceous top-NW thrusting

The results of this study do not confirm the proposed “Intra-Turonian” (Sǎndulescu 1984) age of top-NW thrusting. Based on the combination of sedimentary and geochronological data, we associate ages between 110 and 90 Ma (Albian–Turonian) with NW-directed thrusting of the Dacia Mega-Unit on top of the Tisza Mega-Unit. This age range (90–110 Ma) is also present in age populations of detrital zircon ages from Late Cretaceous syn- to post-tectonic sediments (population 1; Schuller 2004). Post-dating the Alpine metamorphic peak, this Late Cretaceous tectonic phase is responsible for a retrogressive mineral assemblage in lower parts of the nappe stack (chloritization of biotite in the Bihor, Codru and Biharia s.str. nappes). NW–SE-trending stretching lineation and associated NW-directed kinematic indicators (Figs. 4, 6e–g) suggest top-NW-directed thrusting of the Biharia Nappe System on top of the Bihor and Codru Nappes. With the exception of the Vidolm Nappe and the South Apuseni Ophiolites, which escaped reheating during the Late Cretaceous (Kounov and Schmid 2013), reset zircon fission-track data and Rb–Sr biotite ages evidence post-Turonian cooling. However, local shear zones of the Vidolm Nappe exhibit syn-tectonic biotite during the Turonian Phase (sample MR103). Early Cretaceous syn-tectonic sediments of the Meteş-Fm. still record eroded ophiolitic material, albeit from a more distal source area (Suciu-Krausz et al. 2006), whereas Early–Late Cretaceous post-tectonic sediments of the Valea lui Paul-Fm. and basal parts of the Rameţi flysch show a mineralogical change to the erosion of a continental metamorphic source. This change in sedimentation is interpreted to constrain the end of the D2 deformation phase (Suciu-Krausz et al. 2006; Kounov and Schmid 2013, and Fig. 12).

#### D3.1 and D3.2: Late Cretaceous exhumation of the Bihor Unit

Time–temperature paths, fission-track modelling (Kounov and Schmid 2013), and the deposition of syn- to post-tectonic sediments on all but the lowest tectonic units in the nappe stack allow constraining general uplift of the study area during Late Cretaceous times (Fig. 12). The rate and the pattern of this exhumation allow the attribution to extensional unroofing along low-angle detachments. This is further constrained by E-directed mylonitic shear zones (e.g. Fig. 7f), E–W-trending stretching lineations, and E-directed shear bands in the sector C (Fig. 3). Sillimanite gneiss in the Bihor Unit indicates initial phases of post-metamorphic uplift along low-angle detachment zones (Hârtopanu and Hârtopanu 1986). A mylonitic-brittle overprint of steeply dipping fabric elements under greenschist-facies metamorphic conditions (biotite/chlorite isogrades of Hârtopanu and Hârtopanu 1986) can be attributed to extensional exhumation of the Bihor Unit (Pană 1998). Accordingly, the geometries of nappe contacts between the Bihor Unit, the Codru Nappe, and the Biharia Nappe s.str. indicate that partial tectonic omission of the Codru Nappe probably relates to a later brittle stage of this exhumation cross-cutting the ductile detachment (Fig. 3). Thermochronological data indicate rapid cooling of all tectonic units in the study area during Early–Late Cretaceous times (Fig. 11; Kounov and Schmid 2013). Shear bands from structurally higher parts (i.e. the Biharia Nappe s.str.) also evidence E-directed deformation and show the transition to cooler conditions and a subsequent brittle extensional overprint of the nappe contacts. The dominantly NE–SW-trending brittle normal faults are associated with the sedimentation of Upper Cretaceous syn- to post-tectonic “Gosau-type” sediments in the hanging wall. However, where no sedimentary information is available, it is difficult to distinguish structures associated with Late Cretaceous half-graben formation from younger extensional structures associated with the opening of the Transylvanian Basin during the Neogene (Schuller 2004). Although no post-tectonic sediments directly overlie the Bihor and Codru Nappes in the study area, Late Cretaceous sediments were deposited in close vicinity (Fig. 3) and thus allow concluding a structural position close to the surface. Late Cretaceous thermochronological data (Rb–Sr biotite cooling ages and zircon fission-track data), together with the deposition of the “Gosau-type” sediments ($\sim$85–65 Ma; Schuller 2004; Fig. 11a, b), constrain general cooling and surface exposure of the Biharia Nappe System during the Late Cretaceous (Fig. 12). Late Cretaceous uplift and exhumation, post-dating thick-skinned nappe stacking and associated metamorphism in the Apuseni Mountains, concurs with the evolution in the crystalline basement of the Transylvanian Basins, the East- and the South Carpathians (Willingshofer et al. 1999; Krézsek and Bally 2006; Gröger et al. 2013). Gröger et al. (2013) report a previously not recognized phase of presumably E-directed tectonic unroofing (7–11 km) during Albian/Cenomanian times from the Bucovinian Nappe stack of the Maramureş area (Dacia Mega-Unit; East Carpathians). The authors distinguish this exhumation from a second phase of extension and sedimentation during Turonian–Maastrichtian times, which corresponds to the sedimentation of “Gosau-type” deposits in the Apuseni Mountains. Interestingly enough, this Late Cretaceous exhumation associated with syn- to post-tectonic marine sedimentation in the hanging wall (“Gosau-type” sediments) shows significant parallels with the Eastern Alps (Neubauer et al. 1995; Froitzheim et al. 1997; Schuller et al. 2009) and the Dinarides (Gelder et al. 2015).

#### D4: Latest Cretaceous deformation

Our kinematic observations show top-W-directed deformation under brittle conditions (Fig. 7) during the latest compressional stage. These findings are in agreement with the results from several other studies for Late Cretaceous/Early Palaeogene deformation in the study area (Schuller 2004; Merten et al. 2011; Kounov and Schmid 2013). Final cooling to surface temperatures occurred during Cenozoic times (Merten et al. 2011; Kounov and Schmid 2013).

## Conclusion

The integration of sedimentary, thermochronological, and kinematic data allows providing new constraints on timing and kinematics of deformation events during the Alpine tectonothermal evolution of the Apuseni Mountains. New Ar–Ar and Rb–Sr age data constrain differential post-tectonic cooling from medium-grade conditions during Early Cretaceous times. Differences in timing of cooling between the tectonic units relate to their structural position in the nappe stack. The Vidolm Nappe, situated at the highest position of the Biharia Nappe System (Dacia Mega-Unit), shows post-tectonic cooling following the Late Jurassic obduction of the ophiolites, whereas cooling of the Baia de Arieş Nappe is contemporaneous with nappe stacking reported from the Dacia Mega-Unit. The structural data set exhibits complex deformation along E–W- to NE–SW-trending, sinistral transpressive shear zones between the Tisza and Dacia Mega-Units, which precedes penetrative top-NW thrusting in the study area. Top-NW-directed thrusting is responsible for the present-day nappe stack and associated with a more or less pervasive (retrograde) greenschist-facies overprint of the tectonic units in the study area during Early–Late Cretaceous times. Structurally higher parts in the nappe stack, i.e. the Transylvanian Ophiolites, the Vidolm, and Baia de Arieş nappes, were less affected by retrogressive overprinting. Rapid Late–Early Cretaceous cooling in the eastern periphery of the Bihor Unit is associated with E-directed tectonic unroofing in lower structural levels of the Apuseni Mountains. This exhumation takes place along low-angle detachments and exposes structurally lower parts of the basement units. Contemporaneous or slightly delayed upper crustal detachment faulting and associated hanging-wall sedimentation of Late Cretaceous syn- to post-tectonic sediments occurs in the same extensional context. The timing of exhumation agrees with results from other parts of the Dacia Mega-Unit, e.g. the East Carpathians, the South Carpathians, and the Transylvanian Basin, and also allows for a tentative correlation with exhumation in other parts of the Alpine–Carpathian–Dinaride orogenic system.

**Table 1:** Sample locations, mineralogy, and summary of geochronological ages

Sample information					Description				Age (Ma)	
Code	Section	Tectonic Unit	Latitude	Longitude	Lithology	Assemblage	Schistosity	Retrogress. overprint	Rb/Sr Bt	Ar/Ar Ms
MR103	A	Vidolm Nappe	$46^{\circ }30'16.85''$	$23^{\circ }34'45.8''$	Paragneiss	Qz–Ms–Grt-Pl–Bt–Chl–Ap	Strongly deformed	Intermediate	${94.4 \pm 0.9}$	${157.9\pm 0.5}$
MR11	A	Vidolm Nappe	$46^{\circ }30'0.23''$	$23^{\circ }31'44.59''$	Paragneiss	Qz–Bt–Ms–Pl–Grt–Chl–Ky–St–Opq	Undeformed	Weak	${141.3 \pm 1.4}$	278.2 ± 4.2^a^
MR15	B	Baia d. Aries N.	$46^{\circ }27'6.26''$	$23^{\circ }24'31.36''$	Micaschist	Qz–Ms–Fsp–Cal–Chl	Deformed	Intermediate	${80.4 \pm 0.8 }$	**–**
MR66	B	Baia d. Aries N.	$46^{\circ }23'13.0''$	$23^{\circ }18'5.12''$	Paragneiss	Qz–Bt–Ms–Grt–Opq	Undeformed	Weak	${105.8 \pm 1.1}$	**–**
MR150	B	Baia d. Aries N.	$46^{\circ }23'52.66''$	$23^{\circ }13'5.38''$	Paragneiss	Qz–Bt–Ms–Grt–Opq	Undeformed	Weak	${109.0\pm 1.1}$	**–**
MR24	B	Biharia N. s.str.	$46^{\circ }27'47.16''$	$23^{\circ }23'13.24''$	Granite	Qz–Fsp–Ms–Bt–Stp–Chl–Opq	Pressure solution	Intermediate	**–**	${113.3\pm 0.4}$
MR141	C	Codru Nappe	$46^{\circ }37'32.81''$	$23^{\circ }23'28.14''$	Amph. host rock	Hbl (Act)–Ep–Qz–Bt–Chl–Fsp	n.a. (Biotite vein)	Strong	${176.7\pm 1.7}$	**–**
MR113	C	Bihor Nappe	$46^{\circ }38'14.28''$	$23^{\circ }21'50.47''$	Grt-micaschist	Ms–Qz–Grt–Fsp–Bt–Ep–St–Chl–Cld	Strongly deformed	Strong	${80.8\pm 0.8}$	${96.0\pm 0.4}$

Abbreviations of mineral names are according to Whitney and Evans (2010)

^a^Discordant age spectrum

## Electronic supplementary material

Below is the link to the electronic supplementary material.

Supplementary material 1 (xlsx 38 KB)

Supplementary material 2 (xls 96 KB)
